# Therapeutic targets of armored chimeric antigen receptor T cells navigating the tumor microenvironment

**DOI:** 10.1186/s40164-024-00564-w

**Published:** 2024-09-30

**Authors:** Xianjun Li, Tianjun Chen, Xuehan Li, Hanyu Zhang, Yingjing Li, Shuyuan Zhang, Shengnan Luo, Tongsen Zheng

**Affiliations:** 1 Harbin Medical University Cancer Hospital, Harbin, 150081 China; 2 Department of Gastrointestinal Medical Oncology, Harbin Medical University Cancer Hospital, Harbin, 150081 China; 3 Department of Phase 1 Trials Center, Harbin Medical University Cancer Hospital, Harbin, 150081 China; 4 Department of Breast Surgery, Harbin Medical University Cancer Hospital, Harbin, 150081 China; 5 Heilongjiang Province Key Laboratory of Molecular Oncology, Harbin,150081, China

**Keywords:** Chimeric antigen receptor (CAR) T cell, T cell engineering, Cancer immunotherapy, Solid tumor, Tumor microenvironment

## Abstract

Chimeric antigen receptor (CAR) T cell therapy, which targets tumors with high specificity through the recognition of particular antigens, has emerged as one of the most rapidly advancing modalities in immunotherapy, demonstrating substantial success against hematological malignancies. However, previous generations of CAR-T cell therapy encountered numerous challenges in treating solid tumors, such as the lack of suitable targets, high immunosuppression, suboptimal persistence, and insufficient infiltration owing to the complexities of the tumor microenvironment, all of which limited their efficacy. In this review, we focus on the current therapeutic targets of fourth-generation CAR-T cells, also known as armored CAR-T cells, and explore the mechanisms by which these engineered cells navigate the tumor microenvironment by targeting its various components. Enhancing CAR-T cells with these therapeutic targets holds promise for improving their effectiveness against solid tumors, thus achieving substantial clinical value and advancing the field of CAR-T cell therapy. Additionally, we discuss potential strategies to overcome existing challenges and highlight novel targets that could further enhance the efficacy of CAR-T cell therapy in treating solid tumors.

## Background

Chimeric antigen receptor (CAR) T cell therapy involves the genetic modification of T cells to recognize and bind to specific cancer cell surface antigens, thereby enabling them to effectively target and eliminate cancer cells [1]. Since the approval of the first CAR-T cell product targeting the CD19 antigen in B cells by the United States Food and Drug Administration, numerous CAR-T cell products have been developed, demonstrating significant efficacy against hematological malignancies in clinical studies [2].

The differential response of hematological versus solid tumors to CAR-T cell therapy can be attributed to their distinct physical and physiological characteristics [3]. Hematological tumor cells, which are dispersed in the bloodstream, are more readily accessible to CAR-T cells. In contrast, solid tumors are typically encased within a collagen-rich stroma, creating a formidable physical barrier that impedes CAR-T cell infiltration [4]. In addition, the immunosuppressive tumor microenvironment (TME) of solid tumors presents additional challenges by limiting the efficacy of CAR-T cells [5]. Immunosuppression is one of the main features of the TME, promoting the development and growth of tumor cells and hindering the efficacy of CAR-T cells against solid tumors [6].

Various strategies aimed at improving CAR-T cell efficacy in solid tumors by targeting the TME have been proposed, including the combination of CAR-T cell therapy with chemoradiotherapy and TME-targeting drugs [7]. In addition, the development of armored CAR-T cells, engineered to overcome the TME’s barriers, represents a promising approach to improving therapeutic outcomes [8]. In this review, we summarize the current limitations of conventional CAR-T cells related to the TME and the advantages of armored CAR-T cells that directly target the TME. We also discuss the different types and mechanisms of armored CAR-T cells and identify effective therapeutic targets within the TME that could enhance the efficacy of CAR-T cell therapy against solid tumors. Furthermore, we discuss other potential strategies to enhance the capacity of CAR-T cells against the TME in solid tumors and highlight future perspectives in this evolving field. This review systematically summarizes the therapeutic targets of armored CAR-T cells navigating the TME and outlines specific strategies leveraging these targets to enhance the efficacy of CAR-T cell therapy in solid tumors.

## Challenges associated with conventional car-T cell therapy for solid tumors

**Fig. 1:**
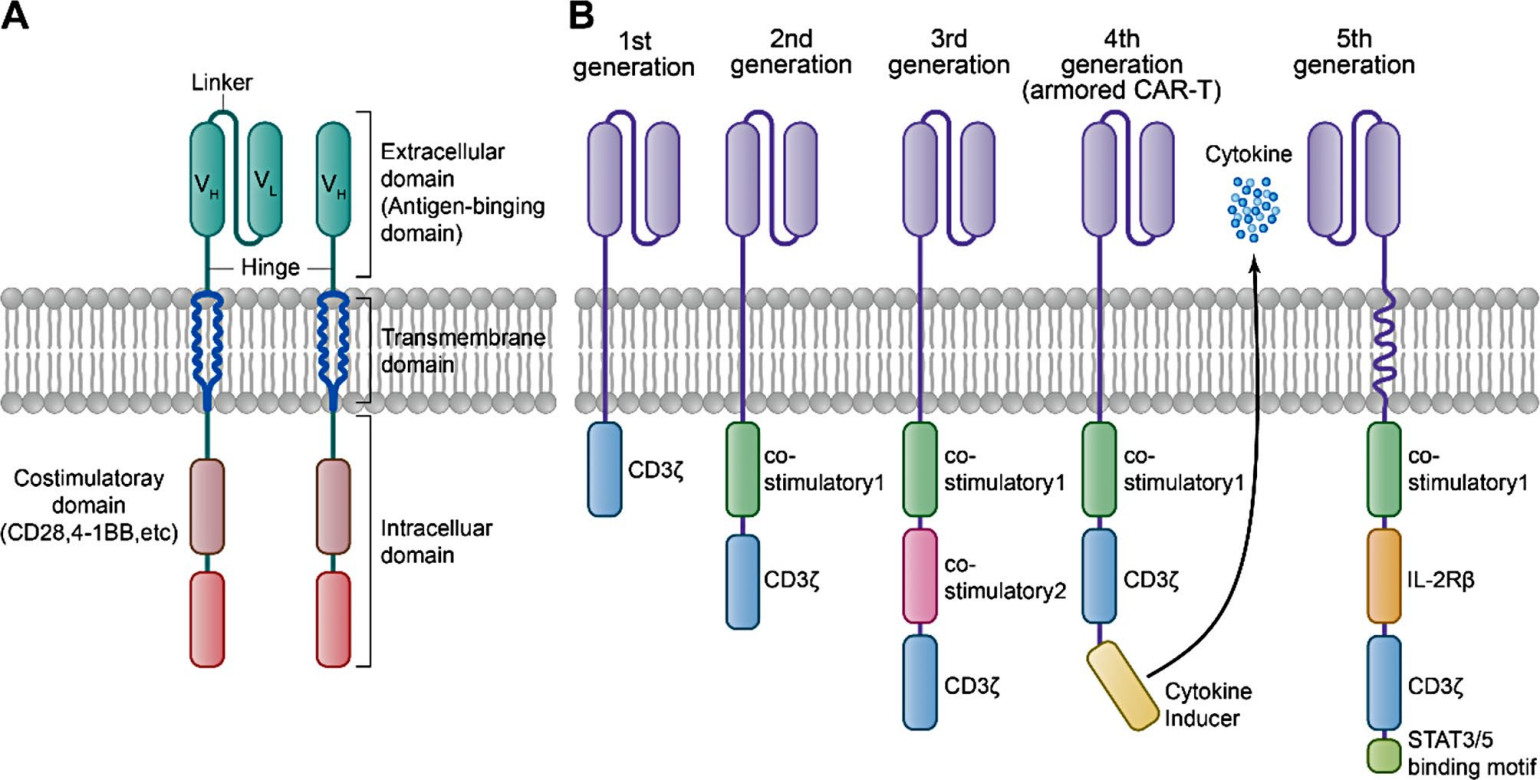
Evolutionary structure of CARs. (**A**) Primary structure of CARs. (**B**) Evolution of the general structure across five generations of CARs. First generation CARs contain only an antigen-recognition domain and a CD3ζ T cell signaling domain. Second generation CARs contain an additional costimulatory domain upstream of the CD3ζ domain. Third generation CARs have a second additional costimulatory domain. Fourth generation CARs (armored CARs) contain an additional secreted cytokine product that is either constitutively or inducible expressed. Fifth generation CARs incorporate an additional cytoplasmic IL-2Rβ domain and carry binding sites for STAT3/5

CARs are typically composed of several key structural components: an antigen-binding domain, a hinge domain, a transmembrane domain, one or more costimulatory domains, and a signal transduction domain (Fig. 1A) [8]. The extracellular antigen-sensing domain of CARs usually consists of single-chain variable fragments (scFvs), derived from the variable regions of the heavy (VH) and light (VL) chains of monoclonal antibodies [9]. Recently, single-domain antibodies composed solely of the heavy chain variable region (VHH), also known as nanobodies, have been increasingly utilized as alternatives to traditional CAR targeting domains. These nanobodies offer significant advantages, including reduced immunogenicity, enhanced stability and specificity, heightened affinity, and a streamlined development process [10]. The hinge and transmembrane regions are typically composed of CD8 or IgG4 proteins, linking the intracellular and extracellular domains of the CAR. The costimulatory domains, primarily made up of CD28 and 4-1BB, activate CAR-T cells, enhancing their proliferation, cytokine secretion, and antitumor activity in vivo [11]. The signal transduction domain generally includes the CD3ζ domain, which transmits extracellular signals that activate T cells, thereby inducing tumor cytolysis and cytokine release [12].

To date, five generations of CARs have been developed, each with updated structures that contribute to enhanced functionality (Fig. 1B) [13]. Despite updates to their structural design, the treatment of solid tumors by CAR-T cells presents several challenges owing to limitations in their mechanisms of action [14]. In the following sections, we discuss the major obstacles faced in this therapeutic approach.

### Lack of suitable targets

Target selection is a crucial determinant of CAR-T cell therapy success [15]. The lack of effective targets in solid tumors represents a significant challenge to the efficacy of this therapeutic approach [16]. Unlike hematological malignancies, where antigen expression is typically more specific in solid tumors—not only between different tumor types but also within individual cells of the same tumor—thereby complicating the process of target selection [17]. Ideally, candidate target antigens, known as tumor-specific antigens, should be exclusively expressed in malignant cells and absent in nonmalignant or healthy tissues. However, many antigens selected for conventional CAR-T cell therapy are tumor-associated antigens that are also expressed, to some extent, in nonmalignant cells. This overlap can lead to serious adverse events, such as on-target/off-tumor toxicity (OTOT), where CAR-T cells inadvertently attack normal tissues [18]. For example, a Phase I trial that used anti-carcinoembryonic antigens (CEA) CAM5 CAR-T cells in patients with advanced CEA CAM5-positive solid tumors resulted in severe OTOT, causing clinically severe adverse events such as respiratory distress [19]. In recent years, neoantigens have emerged as promising tumor-specific targets for oncology treatments, including CAR-T cell therapy. Neoantigen vaccines have shown success in treating patients with melanoma by expanding neoantigen-reactive T cells that can potentiate stronger antitumor responses [20]. However, this neoantigen-based approach remains challenging in tumors with a low mutation burden, as only a small fraction of mutations produce neoantigens with sufficient reactivity to be effective for tumor therapy [21].

### Suboptimal persistence

Due to the presence of T cell exhaustion and peripheral tolerance, CAR-T cells exhibit suboptimal persistence in vivo [22]. Peripheral tolerance refers to a state where T cells recognize antigens but fail to mount a responsive attack against cancer cells [23]. Elevated cytokine levels may be associated with immune tolerance. The TME of different tumors may be characterized by varying cytokine profiles, affecting the development of immune tolerance [24]. Various cytokines, including interleukin 6 (IL-6), IL-1, and interferon-γ (IFN-γ), are involved in this process and contribute to the suboptimal persistence of CAR-T cells together [25]. Compared to unmodified natural T cells, CAR-T cells experience more continuous antigenic stimulation. Due to chronic antigen exposure, CAR-T cells may develop dysfunctional characteristics similar to those of endogenous exhausted T cells, which are characterized by reduced expression of effector molecules and elevated expression of inhibitory immune checkpoint molecules such as programmed cell death protein 1 (PD-1), lymphocyte-activation gene 3 (LAG-3), and T cell immunoglobulin domain and mucin domain 3 (TIM3) [26].Additionally, CAR-T cells encounter metabolic challenges in solid tumors. Metabolically active cancer cells deplete essential nutrients like glucose, amino acids, and fatty acids from the TME, limiting their availability to CAR-T cells [27]. Furthermore, the accumulation of metabolic byproducts such as adenosine and reactive oxygen species (ROS), produced by tumors, contributes directly to immune suppression, impairing CAR-T cell function. Excessive lactate production within the TME also hinders CAR-T cell-mediated antitumor responses, leading to premature exhaustion and reducing their persistence in solid tumors [28].

### Immunosuppressive TME

The physical barriers and immunosuppressive effects within the TME are major mechanisms by which solid tumors evade CAR-T cell-mediated killing [18]. Research indicates that stromal cells, such as cancer-associated fibroblasts (CAFs), are activated by transforming growth factor beta (TGF-β), which stimulates the production of extracellular matrix (ECM) proteins, thereby impeding CAR-T cell motility [29]. TGF-β can also directly affect CAR-T cells by suppressing the expression of chemokine receptors like C-X-C chemokine receptor type 3 (CXCR3), limiting their ability to infiltrate solid tumors [30]. Additionally, abnormal vascular structures within solid tumors often reduce levels of adhesion molecules essential for CAR-T cell entry, such as VCAM1 and ICAM1, further hindering CAR-T cell infiltration [16]. Moreover, solid tumors commonly express high levels of immune checkpoint molecules, including PD-L1 and cytotoxic T-lymphocyte-associated antigen 4 (CTLA-4), which bind to receptors on CAR-T cells to inhibit their activation and cytotoxic functions [31]. Tumors also release immunosuppressive cytokines such as IL-10 and TGF-β, further suppressing CAR-T cell activity [27]. Tumor-associated macrophages (TAMs), regulatory T cells (Tregs), and myeloid-derived suppressor cells (MDSCs) in the TME secrete suppressive factors or interact directly with CAR-T cells, impairing their efficacy [32]. TAMs, the most abundant immune-infiltrating cells within the TME, suppress T cell-mediated antitumor immunity by secreting cytokines and amino acid-depleting enzymes such as arginase 1 and indoleamine 2,3-dioxygenase (IDO), as well as promoting the recruitment of Tregs [33]. Tregs specifically hinder cytotoxic T cell function by releasing immunosuppressive cytokines, competitively consuming IL-2, suppressing antigen-presenting cells (APCs) through CTLA-4-mediated inhibition, and blocking T cell activation [34]. MDSCs, known for their potent immunosuppressive capabilities, directly target effector T cells, significantly reducing CAR-T cell efficacy [35]. Consequently, even if CAR-T cells successfully infiltrate tumor tissues, their effector functions are often significantly diminished, severely compromising their ability to kill tumor cells.

Moreover, solid tumors typically exhibit high metabolic activity, consuming substantial amounts of glucose, oxygen, and other nutrients, which creates a nutrient-deprived local microenvironment. This resource competition impacts CAR-T cell proliferation and function [36]. Elevated lactate levels, produced by hypermetabolic tumor cells, are associated with NFAT-mediated dampening of T cell signaling, expansion of Tregs, and polarization of macrophages toward an immunosuppressive M2 phenotype [37]. Abnormal vasculature induces tissue hypoxia, which not only recruits immunosuppressive cells by secreting various chemokines but also upregulates the expression of CTLA-4 and LAG-3 proteins on Tregs and PD-L1 on MDSCs, TAMs, and tumor cells, further impacting CAR-T cell infiltration and effector function [38].

### Immune evasion

Immune escape refers to the phenomenon where tumor cells proliferate by evading the monitoring, recognition, and attack of the body’s immune system by altering their own surface antigens and recruiting suppressive immune cells and molecules, which presents significant challenges to the effectiveness of immune cell therapies, including CAR-T cell therapy [39].The genetic and phenotypic heterogeneity of antigens is common among different solid tumors and even within individual cells of the same tumor. Low or absent antigen expression can prevent CAR-T cells from achieving the activation threshold necessary for effective recognition, leading to immune escape and reduced targeting efficiency and cytotoxicity in solid tumors [40]. Furthermore, the selective pressure exerted by CAR-T cells can prompt tumors to adopt evasion mechanisms such as reducing antigen expression or complete antigen loss. This results in antigen levels falling below the threshold required for CAR-T cell activation, ultimately leading to tumor progression through immune escape. Additionally, during CAR-T cell therapy, increased secretion of IFN-γ can induce the upregulation of immunosuppressive molecules like PD-L1 on the tumor surface as a response to host immune pressure. PD-L1 binds to PD-1 receptors on T cells, transmitting inhibitory signals that inactivate T cells and promote tumor progression [41]. The binding of PD-1 to PD-L1 inhibits phosphorylation events in T cells, disrupting T cell receptor signaling and diminishing T cell activity. This inhibition can lead to cell cycle arrest and even trigger apoptosis in T cells, depriving them of their ability to kill tumor cells and facilitating immune escape. Additionally, activation of the PD-1/PD-L1 pathway not only impairs tumor immunity but also inhibits T cell migration and infiltration into the tumor site, further reducing the efficacy of CAR-T cells by limiting the presence of effective T cells within the TME.

## Major cells in solid tumors and their role in TME regulation

**Fig. 2:**
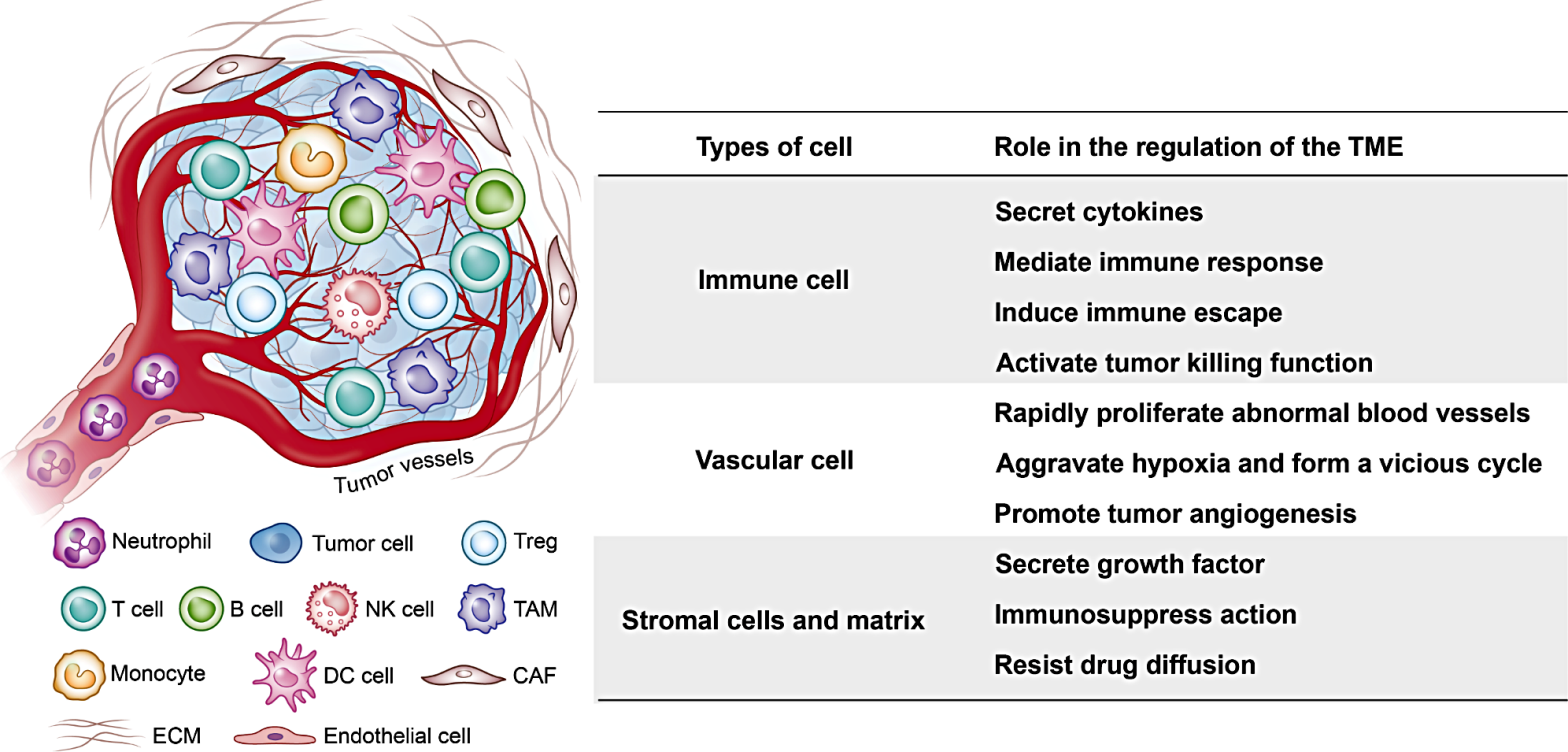
Cellular components and their roles in the regulation of the TME

The TME in solid tumors includes various cells such as immune, vascular, and stromal cells, which play different roles in TME regulation (Fig. 2) [6].

### Immune cells

Solid tumors host a substantial number of adaptive immune cells, such as T cells and B cells, and they play both dual roles of inhibiting tumor progression and promoting tumor growth in TME. For example, B cells exert antitumor effects through mechanisms like antibody-dependent cell-mediated cytotoxicity (ADCC) and complement activation. However, they may also foster tumor growth by secreting anti-inflammatory and pro-angiogenic mediators that induce immunosuppression [42]. T cells are broadly categorized into two main subpopulations: CD4^+^ and CD8^+^ T cells. CD8^+^T cells are key effector cells in the antitumor immune response, capable of promoting tumor growth by binding to T cell receptors on the surface of the tumor cells, by apoptosis mediated through granzymes and perforin, or by FASL-FAS-mediated cell death that disrupts target cell-specific recognition and kills cancer cells. CD4^+^helper T cells support CD8^+^T cells in mounting an immune response. Th1 and Th2 are the most common CD4^+^T cell subtypes; they secrete different cytokines upon activation, thus playing contrasting roles in solid tumors. Th1 subtype of CD4^+^T cells directly destroy tumor cells by secreting IFN-γ and TNF, whereas Th2 subtype secretes anti-inflammatory mediators such as IL4, which suppress immunity and support tumor growth. Tregs are another highly immunosuppressive subpopulation of CD4^+^T cells, which can dampen T cell-mediated antitumor immune responses by releasing inhibitory cytokines (e.g., IL-10 and TGF-β) and by competing with effector T cells for IL-2, thus depleting this critical growth factor [34].

Myeloid immune cells in solid tumors also play an important role in regulating the TME. TAMs are immune cells that can either promote or inhibit tumor growth and are classified as either M1-like or M2-like [43]. M1-like macrophages release pro-inflammatory factors such as ROS, nitric oxide, and IL-6, effectively presenting antigens and activating a strong immune response against the tumor [44]. Conversely, M2-like macrophages are activated by IL-4 and IL-10 and release anti-inflammatory cytokines that dampen the immune response while promoting angiogenesis and tissue repair. Typically, M2-like macrophages support tumor growth, whereas M1-like macrophages can inhibit it [45]. Dendritic cells (DCs), originating from bone marrow hematopoietic stem cells, are divided into classical myeloid cells (cDCs) and plasmacytoid dendritic cells (pDCs). Both subtypes exhibit strong antigen-presenting capacities and express cytokines required for activating CD4^+^ or CD8^+^T cell functions [46]. However, in the TME, the expression of DC surface costimulatory molecules is often inhibited or downregulated, which impairs their ability to uptake, process, and present antigens, thus contributing to the expression of Major histocompatibility complex I (MHC I)/MHC II class I molecules, leading to DC-associated tumor immune escape [47]. Neutrophils, the most abundant immune cells in the blood, exhibit dual roles in tumor dynamics based on their polarization status. They can either inhibit tumor growth by killing tumor cells through the release of nitric oxide synthase, or ROS, or promote tumor growth by facilitating angiogenesis and the dissemination of tumor cells within the TME [48]. Natural killer cells (NKs) are innate lymphoid-like cells with notable cytotoxic capabilities, particularly effective in recognizing and eliminating stressed cells lacking MHC I-like expression [49], thereby exerting a potent anticancer function. Monocytes, circulating in the blood and capable of differentiating into macrophages and DCs within tissues, exhibit both pro- and antitumor activities. They secrete anticancer mediators and stimulate functional activation of NKs, but within the TME, they may support pro-tumorigenic immune functions by remodeling the extracellular matrix and inducing angiogenesis [50]. MDSCs consist of immature mononuclear MDSCs and polymorphonuclear MDSCs, both highly immunosuppressive and capable of inhibiting the effector functions of T cells, NKs, and DCs, thereby allowing tumors to evade immune surveillance [51].

### Vascular cells

The structure of the tumor vasculature is disturbed. The extreme imbalance between pro- and anti-angiogenic factors results in the rapid generation and abnormal proliferation of vessels in the TME, which are typically distorted, unevenly distributed, and dysfunctional [52]. Tumor vasculature endothelial cells (ECs) are essential molecules in the abnormal vasculature of tumors that promote tumor progression and exhibit a high degree of heterogeneity and plasticity [53]. In addition, the permeability of the abnormal tumor vasculature is increased by the shedding of pericytes surrounding vasculature ECs, resulting in protein leakage into the vasculature and increased interstitial fluid pressure in the TME [54]. The increased interstitial fluid pressure compresses the vasculature and exacerbates hypoxia, which in turn induces tumor angiogenesis, creating a vicious cycle [55].

### Extracellular matrix and stromal cells

ECM is primarily composed of collagen and laminin, providing essential support for cell communication, proliferation, and adhesion [56]. As a noncellular component, the ECM offers structural support and biochemical factors that are favorable to cancer cell survival and growth [57]. Within the TME, the ECM significantly impacts T cell infiltration. While providing structural support to tumor cells, the ECM also inhibits T cell penetration and effectiveness through physical barriers and signaling mechanisms [58]. The density and complex composition of the ECM can obstruct the migration of CAR-T cells, hindering their ability to reach and infiltrate the tumor core, thus limiting their antitumor activity. The ECM in the TME not only facilitates the growth, invasion, and metastasis of tumor cells but also promotes tumor angiogenesis and impedes drug diffusion [59]. Additionally, in the TME, the ECM interferes with tumor antigen presentation, influencing the effectiveness of effector T cells in attacking cancer cells [60]. CAFs play crucial roles in the tumor stroma. These cells are highly active and secrete various cytokines that promote the formation of new blood vessels within the tumor, facilitating the transformation of tumor cells and disrupting the cellular balance in the tissue [61]. Despite sharing some characteristics with normal fibroblasts, CAFs are more dynamic and grow faster than them [62]. Furthermore, CAFs produce high levels of cytokines, matrix proteins, and immune-regulating factors that not only promote tumor growth but also help tumor cells evade immune responses, thereby enhancing metastasis and contributing to treatment resistance [63]. Given the critical roles of the ECM and stromal cells in maintaining the TME, their regulation is essential for improving the efficacy of tumor immunotherapy [64].

## Armored car-T cells may overcome the TME barrier

### Types and mechanisms of armored CAR-T cells

**Fig. 3:**
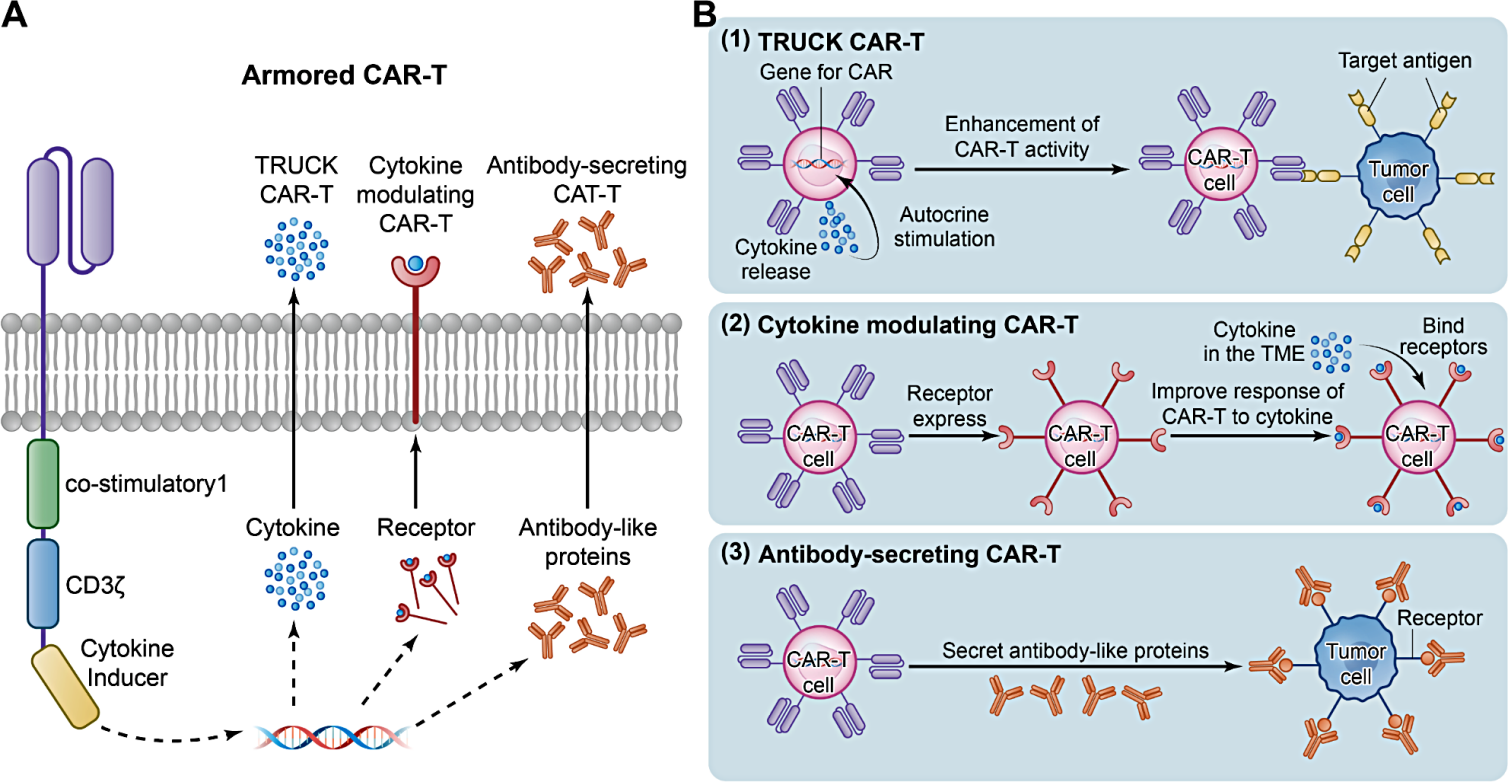
Structures and mechanisms of three types of armored CAR-T cells. (**A**) The three different types of armored CAR-T cells: TRUCKs, cytokine modulating CAR-T cells, and antibody-secreting CAR-T cells. (**B**) Mechanisms of the three types of armored CAR-T cells

Armored CAR-T cells can be divided into three types: T cells redirected to universal cytokine killing (TRUCK) CAR-T cells, cytokine-modulating CAR-T cells, and antibody-secreting CAR-T cells (Fig. 3A) [65].

#### TRUCK CAR-T cells

TRUCK CAR-T cells can help target solid tumors by releasing cytokines such as IL-12 and IL-18 [66]. Engineered to regulate the cytokine environment in the TME, they can enhance the antitumor effects of CAR-T cells and resident immune cells (Fig. 3B).

For instance, Tamada et al. enhanced the antitumor potential of CAR-T cells by introducing IL-7 and C-C chemokine ligand 19 (CCL19) genes to produce 7 × 19 CAR-T cells. These cells not only helped DCs and T cells to infiltrate tumor tissues but also achieved complete tumor elimination in various solid tumor models, including lung and pancreatic cancers [67]. More recently, Tang et al. engineered metabolically enhanced CAR-T cells that autocrine IL-10. These cells promote their own proliferation and effector functions in vivo via IL-10 expression and enhance oxidative phosphorylation metabolism in the TME. In diverse tumor models, including melanoma, breast cancer lung metastasis, subcutaneous colon cancer, and an in situ model of human pancreatic cancer in NSG mice, these CAR-T cells successfully achieved complete tumor regression and were able to resist secondary tumor cell injection, providing durable immune protection to the organism [8].

#### Cytokine-modulating CAR-T cells

Autocrine cytokines enhance the antitumor function of armored CAR-T cells [8], and manipulating the response of CAR-T cells to cytokines is another potential strategy to improve the efficacy of CAR-T cells against solid tumors (Fig. 3B). Based on this, cytokine-regulated CAR-T cells have been generated [68]. Carl June and his team demonstrated that the antitumor activity of T cells can be enhanced by a modified IL-2 cytokine receptor platform through specific stimulation of o9R signaling in CAR-T cells. This modification maintained superior tumor suppressor activity in pancreatic cancer models, even in the absence of the rigors of chemo-/radiotherapy [69]. Additionally, the use of armored CAR-T cells reduces immunosuppression in the TME by down-regulating the activity of inhibitory cytokines [70]. This modification allows cytokines to bind to receptors and be “sequestered” before activating downstream signaling pathways. As a result, CAR-T cell proliferation in the TME is increased, and exhaustion is prevented [71]. In addition, excessive cytokine production can trigger a systemic inflammatory response, leading to adverse events such as cytokine release syndrome. Cytokine-modulated CAR-T cells can enhance the efficacy of CAR-T cells while inhibiting negative cytokines, thus improving the safety of CAR-T cells [72]. Stephen et al.. enhanced CAR-T cells by incorporating modular chimeric cytokine receptors for IL-10 and IL-12. This innovation enables CAR-T cells to thrive in cytokine-deficient environments and improve their efficacy in treating solid tumors, such as brain tumors. This design effectively minimizes cytokine-associated toxicity and provides essential signals that allow CAR-T cells to function effectively in the suppressive TME [68].

#### Antibody-secreting CAR-T cells

Antibody-secreting CAR-T cells leverage the tumor-targeting capabilities of CAR-T cells to deliver secreted antibodies directly to the tumor site. These antibodies synergize with CAR-T cells to enhance antitumor effects while minimizing associated toxicities (Fig. 3B) [73]. The research team led by Renier J. Brentjens engineered T cells to express a CAR that recognizes tumor antigens and concurrently express the scFv segment of a PD-1 antibody. This design blocks the interaction between immune cells PD-1 and tumor cells PD-L1, effectively deactivating PD-1-mediated immunosuppression [74]. Unlike the systemic distribution seen with PD-1 monotherapy, these antibody-secreting CAR-T cells deliver a precise attack on tumors by secreting “PD-1 scFv” directly at the tumor site. This targeted approach avoids toxic side effects at non-tumor sites and has demonstrated promising antitumor effects in various cancers, including ovarian cancer and lymphoma. However, bispecific antibodies face limitations in clinical applications due to their short half-lives and lack of specific targeting. Addressing these challenges, Huang et al.. designed a CAR-T cell targeting CEA that secretes PD-1-TREM2 bispecific antibodies within colorectal tumor tissue. This approach leverages the localized presence of CAR-T cells at the tumor site to extend the in vivo half-life of the bispecific antibodies [33]. The PD-1-TREM2 scFv not only inhibits the PD-1/PD-L1 signaling axis but also blocks the binding of ligands to the TREM2 receptor on MDSCs and TAMs, thereby reducing the ratio of MDSCs to TAMs, significantly enhances the effector function of CAR-T cells, and improves therapeutic efficacy in colorectal cancer.

### Advantages of armored CAR-T cells targeting the TME

#### Boosting CAR-T cell infiltration

Studies have shown that collagen fibers within the ECM surrounding tumors can restrict T cell entry into the TME [31]. Therefore, degrading the ECM to facilitate T cell infiltration may represent a promising strategy to enhance the efficacy of CAR-T cell therapy. For instance, Coruana et al.. engineered CAR-T cells to express Heparanase (HPSE), an enzyme capable of degrading heparan sulfate proteoglycans, thereby enhancing their ability to degrade the ECM and significantly improving their infiltration and antitumor activity in stroma-rich solid tumors [29]. Additionally, leveraging the matrix metalloproteinase (MMP)-secretion capability of macrophages represents another strategy. Since MMPs regulate the synthesis and degradation of ECM components, enhancing their activity can remodel the ECM and promote T cell infiltration into tumors. This approach has shown promising antitumor effects in breast cancer models [75].

Chemokines are key molecular factors determining the extent of cytotoxic T cell infiltration in solid tumors. In many solid tumors, the chemokine expression profile tends to favor immunosuppressive cell types [76]. By designing armored CAR-T cells that express chemokine receptors, which recruit immunocompetent cells and target the upregulated chemokines in the TME, it may be possible to overcome the abnormal chemokine landscape of the TME, thereby enhancing T cell infiltration and improving the efficacy of CAR-T cell therapy against solid tumors [77]. For example, L. Cadilha et al.. developed C-C chemokine receptor 8 (CCR8)-DNR-CAR-T cells by combining CCR8 with a dominant-negative TGF-β receptor (DNR), enhancing tumor targeting and extending CAR-T cell persistence. They found that CCL1, secreted by activated T cells, provides positive feedback for the recruitment of CCR8 T cells to the tumor tissue, thereby increasing CAR-T cell infiltration and synergizing with DNR to mitigate tumor-derived immunosuppressive signals. In a mouse model of pancreatic solid tumors, CCR8-DNR-CAR-T cells targeting murine EpCAM achieved a tumor rejection rate in three out of seven mice [78]. Additionally, CAR-T cells can be genetically engineered to express chemotactic factors that enhance tumor infiltration. Adachi et al.. designed CAR-T cells producing IL-7 and CCL19, significantly boosting their antitumor potential while promoting the infiltration of DCs and T cells into tumor tissues. This approach achieved complete regression of pre-established P815-hCD20 tumors and extended the survival in mice [67]. Subsequently, Zhang et al.. reported results from a Phase I clinical trial utilizing these IL-7 and CCL19 co-expressing (7 × 19) CAR-T cells, demonstrating robust antitumor effects in Glypican-3 (GPC3)-positive hepatocellular carcinoma and MSLN-positive pancreatic cancer [79].

#### Resisting immunosuppression in the TME

The TME in solid tumors suppresses the activation and proliferation of CAR-T cells through its immunosuppressive cells (such as Tregs, MDSCs, and TAMs), cytokines, and immune checkpoint molecules like CTLA-4 and PD-1, thereby impairing the antitumor efficacy of CAR-T cells [80]. Unlike traditional CAR-T cells, armored CAR-T cells equipped with specific receptors can target inhibitory components within the TME, thereby remodeling the TME and enhancing the effectiveness of CAR-T cell therapy in solid tumors [81]. The research team led by Carl H. June previously demonstrated that overexpressing a dominant-negative mutant of the TGFβR II gene in PSMA-targeting CAR-T cells significantly inhibited TGF-β signaling within the tumor, thereby greatly enhancing the antitumor efficacy of CAR-T cells [70]. Recently, they published results from a Phase I clinical trial showing that PSMA-targeting CAR-T cells co-expressing TGFβRDN were safe and effective in treating metastatic castration-resistant prostate cancer and significantly promoted CAR-T cell proliferation in vivo [82]. Additionally, the research team led by Professor Dayenne G. van Leeuwen demonstrated in multiple in vivo models that CAR-T cells expressing PD-1 scFv continuously released PD-1 scFv within the TME, effectively blocking the PD-1/PD-L1 inhibitory axis. This approach not only significantly prolonged the survival of mice but also showed better efficacy than that achieved by combined CAR-T cells with PD-1 antibodies while reducing drug-related toxicities [74]. Moreover, CAR-T cells can be engineered to secrete pro-inflammatory cytokines, thereby remodeling the TME and supporting their own antitumor functions. For example, CAR-T cells that secrete IL-12 or IL-18 can recruit inflammatory M1-like macrophages to the TME, enhance IFN-γ secretion, inhibit Treg proliferation, and prevent immune suppression within the TME [83, 84].

#### Improved persistence

Under normal immune conditions, cytokines play a crucial role in regulating inflammation [85]. Armored CAR-T cells engineered to target cytokines within the TME can effectively counteract the immunosuppressive effects on immune cells. Through autocrine and paracrine mechanisms, cytokine-expressing armored CAR-T cells can transform “cold” tumors into “hot” tumors, enhancing their responsiveness to immune attack [86]. Moreover, armored CAR-T cells expressing cytokines can improve the persistence and efficacy of cell therapy against solid tumors.

A positive correlation has been observed between cytokine concentrations and the activity level of cytokine-expressing armored CAR-T cells, potentially improving treatment accuracy and effectiveness [87]. Cytokines secreted by CAR-T cells could promote the formation of memory T cells, which can activate host T cell responses while supporting the persistence of CAR-T cells [88]. By transfecting mRNA encoding IL-12 and IL-18 into T cells, Melero et al. developed a CAR-T cell capable of autocrine these two cytokines with enhanced antitumor ability, which significantly inhibited the proliferation of tumor cells in melanoma and demonstrated good antitumor activity and durability [86]. Furthermore, CAR-T cells armored with specific mechanisms for navigating the TME can precisely target antigens, reducing the likelihood of off-target effects, thereby limiting toxicity and enhancing CAR-T cell persistence [18]. Recently, Yang et al. achieved significant advances by expressing SMAD7, a negative regulator of the TGF-β/SMAD signaling pathway, in CAR-T cells. In a HER2^+^HeLa cell solid tumor model, this modification not only effectively reduced the inhibitory effects of TGF-β on CAR-T cells, enabling sustained antitumor activity, but also significantly decreased the production of inflammatory cytokines, thereby improving the safety profile of CAR-T cells in vivo [89].

#### Overcoming antigenic heterogeneity

Unlike the typically singular and highly specific targets found in hematological malignancies, solid tumors exhibit significant antigen heterogeneity. This diversity presents a substantial challenge for CAR-T cell therapy, as it complicates the complete eradication of all tumor cells, allowing some tumor cells to evade immune attack. This increases the risk of treatment failure and relapse [17, 90]. To address these challenges, researchers have developed armored CAR-T cells. These cells are designed to enhance antigen recognition and incorporate multi-target strategies to overcome the limitations posed by antigen heterogeneity and improve therapeutic efficacy.

Antigen-specific recognition is pertinent for effective CAR-T cell therapy. In solid tumors, the most commonly targeted tumor-associated antigens (TAAs) include CEA, HER2, GPC3, and EpCAM, whereas tumor-specific antigens (TSAs) are relatively rare, which significantly limits the application of CAR-T cell therapy in solid tumors. In response, researchers are not only continuing to develop CAR-T therapies targeting TSAs but are also focusing on modifying CAR-T cells to enhance their ability to recognize tumor antigens.

Unlike traditional CAR-T cells that directly target the tumor cells, researchers have proposed a new concept: modular CAR-T cells (mod CAR-T). These CAR-T cells typically work in conjunction with adaptors, where one end of the adaptor binds to the CAR-T cell and the other end recognizes and binds to tumor surface antigens. This approach not only increases antigen-recognition specificity, reducing the likelihood of tumor escape, but also allows for the control of T cell activation states by manipulating the adaptors, thus achieving a dual effect [91]. Additionally, synthetic Notch (synNotch) receptors, upon recognizing surface ligands, can trigger the expression of target genes [92]. For instance, Wendell et al.. have incorporated the synNotch system into CAR-T cells, enabling the T cells to regulate the expression of relevant TAAs once they migrate to the tumor site. This adjustment prevents attacks on normal tissues and significantly increases the specificity of antigen recognition by CAR-T cells [93], while also establishing a foothold in the target tumors. This presence is bolstered by synthetic Notch-induced IL-2 production, initiating CAR-mediated T cell expansion and cytotoxic activity [93]. Building on this, Jason and colleagues developed “universal” receptor systems where receptor specificity can be directed post-translationally via the covalent attachment of a co-administered antibody bearing a benzylguanine motif. This expands the targeting capabilities of synNotch receptors. They demonstrated that SNAP-CAR and SNAP-synNotch receptors can be successfully activated by clinically relevant BG-conjugated antibodies, inducing antitumor activity in vivo in a human tumor xenograft mouse model [94].

Dual-specific CAR-T cells, which target two antigens, have been effectively utilized in treating hematologic cancers. This approach not only enhances the antigen-recognition capabilities of CAR-T cells but also boosts their therapeutic efficacy and safety while reducing the risk of tumor escape [95]. Similarly, CAR-T therapies targeting multiple TAAs are emerging as a crucial strategy in the treatment of solid tumors.

Compared to single-specificity CARs, modifying multi-target CARs to recognize multiple tumor antigens can more effectively eliminate established tumors. For example, Choi et al.. developed an innovative dual-specific tandem CAR-T (TanCART) cell designed to simultaneously target both EGFRvIII and IL-13Rα2, two well-known tumor antigens commonly present on the surface of GBM cells yet entirely absent from normal brain tissues. The TanCAR technology enables independent recognition of each antigen and activation of the T cells. However, its function is synergistically enhanced when both antigens are simultaneously recognized together [96]. Similarly, CAR-T cells targeting both CD19 and HER2 have demonstrated enhanced antitumor activity in vivo compared to single-target CAR-T cells [97]. Additionally, trivalent CAR-T cells targeting HER2, IL13Rα2, and EphA2 have effectively eliminated tumor cells [98]. Moreover, targeting both GD2 and B7-H3, two NB-associated antigens, while providing CD28 and 4-1BB co-stimulation, resulted in rapid and sustained antitumor effects in mice and prevented tumor immune escape due to low antigen density [99]. However, the combination of multiple targets increases the potential for off-target effects in CAR-T cells, and constructing multi-target CARs significantly increases the genetic payload delivered to cells, which can lead to reduced transduction efficiency [100]. Therefore, further research is needed to improve the design and safety of multi-target CAR-T cells.

## Current effective targets of armored car-T cells navigating the TME

### Targeting TAMs

**Fig. 4:**
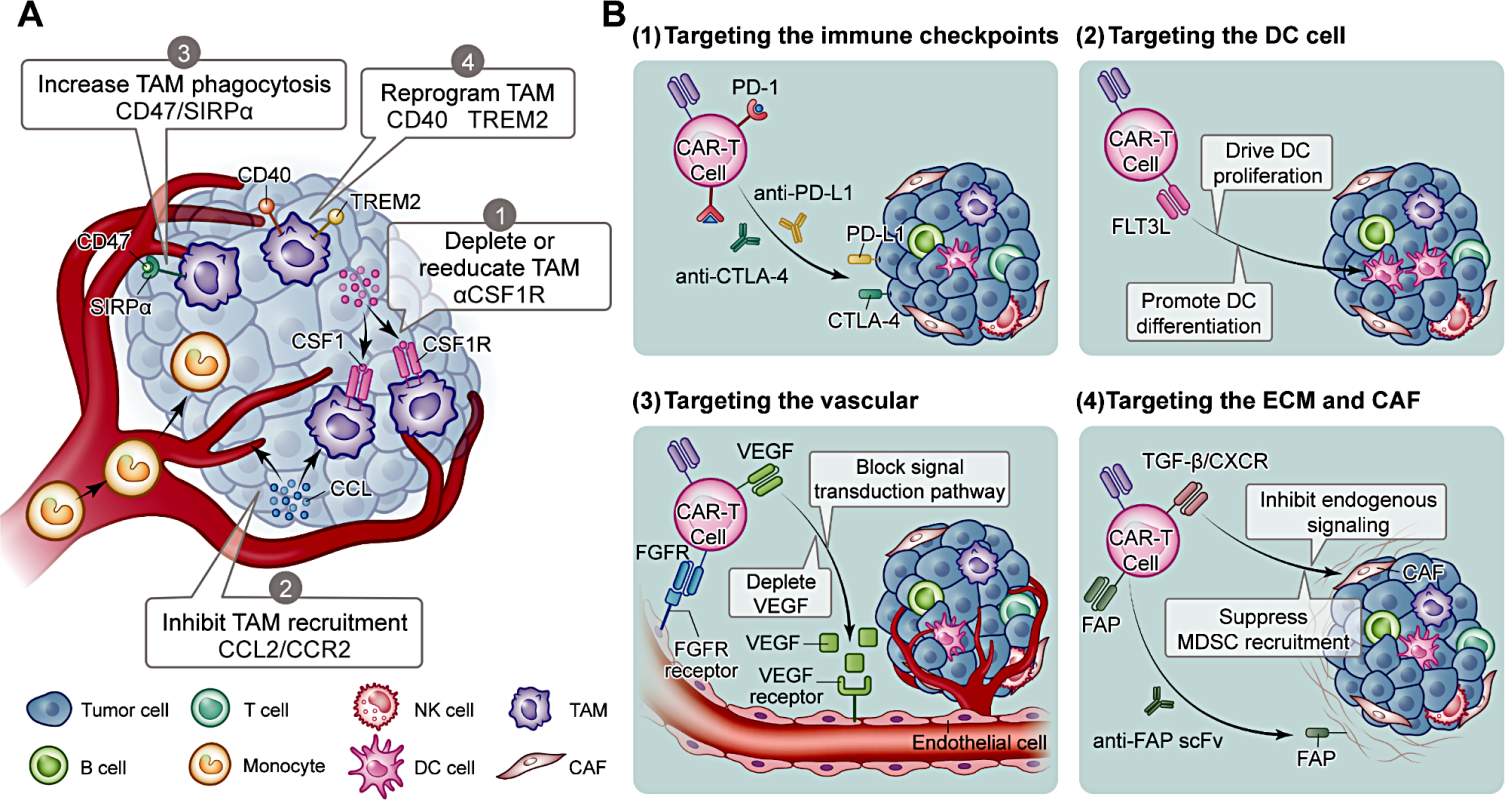
Strategies for armoring CAR-T cells with specific targets for navigating the TME. (**A**) Four main strategies are used for targeting TAMs in the TME. (1) Depletion or reeducation of TAMs. (2) Inhibition of TAM recruitment by targeting the CCL2/CCR2 axis. (3) Increase of TAM-mediated phagocytosis by targeting and inhibiting the CD47/SIRPα signaling axis. (4) Reprograming of the TAM phenotype by inhibiting TREM2 or targeting CD40. (**B**) Strategies for targeting different components in the TME. (1) Targeting the immune checkpoint. (2) Targeting DCs. (3) Targeting the tumor vasculature. (4) Targeting the ECM and CAFs. CCL2, C-C chemokine ligand 2; CCR, C-C chemokine receptor 2; SIRPα, CD47–signaling regulatory protein α; TREM2, triggering receptor expressed on myeloid cell 2

Macrophages are present in most tissues and help regulate the immune response and tissue homeostasis [101]. During tumorigenesis, the macrophage pool creates a distinct subset of TAMs, corresponding to a distinct tumor phenotype and functional heterogeneity [102]. In the TME, TAMs can exhibit pro- or anti-inflammatory functions, showing some plasticity in the regulation of tumor progression [103]. Moreover, their abundance is typically associated with treatment efficacy and patient prognosis, demonstrating their potential as therapeutic targets (Fig. 4A) [104].

Armored CAR-T cells targeting TAMs in the TME not only reduce the ability of TAMs to promote cancer cell survival but also increase CD8^+^T cell cross-presentation and T cell immune reactivity [105].

#### Colony-stimulating factor 1 receptor

The colony-stimulating factor 1 receptor (CSF1R) is a class III receptor for transmembrane tyrosine kinases in macrophages that plays an essential role in macrophage differentiation and survival [106]. The CSF1R signaling pathway is activated by its ligands, CSF1 and IL-34. Notably, elevated serum CSF1 levels are highly correlated with low survival rates in patients with ovarian and endometrial cancer [107]. Yang et al. successfully established a third-generation CAR (A3-CAR) that specifically targets human CSF1R. They generated A3-CAR-T cells from A3-CAR constructed in normal peripheral blood T cells and found that these cells had better target specificity than regular CAR-T cells [108]. Notably, these A3-CAR-T cells were expected to precisely kill and mitigate the inhibitory effects of M2-like TAMs in the TME without causing cytotoxicity to monocytes in human peripheral blood, offering a promising new approach for CAR-T therapy to overcome immunosuppression in solid tumors.

#### CCL2/CCR2 axis

CCL2 is a C-C chemokine ligand produced mainly by activated T cells and plays an important role in tumor progression by activating the host pro-tumor phenotype after binding to the classical CCR2 [109]. Cancer cells release CCL2 into the circulation, which recruits tissue macrophages and CCR2-expressing Ly6Chi monocytes to the tumor site, where they differentiate into TAMs, thereby increasing the number of TAMs within the TME [110]. Treatment of murine cancer models with inhibitory CCL2 antibodies controlled tumor progression; however, once treatment was discontinued, the survival rate of mice declined, highlighting the potential of drugs targeting CCL2 in the treatment of solid tumors [111]. Foster et al. designed a GD2-specific CAR-T cell expressing CCR2b using a GD2-targeting CAR for neuroblastoma. These CAR-T cells, which modulate the CCL2/CCR2 axis, demonstrated enhanced antitumor activity compared to cells not expressing CCR2b [111]. Du et al. employed an engineering strategy to overexpress CCR2 in B7-H3-targeting CAR-T cells, significantly enhancing their ability to traverse the blood-brain barrier, thereby improving the efficacy of adoptive T cell therapy in patients with brain metastases from solid tumors [112].

#### CD47–signaling regulatory protein α (SIRPα)

In the TME, TAMs exhibit distinct phenotypes with varying effects on immune system regulation. TAM phagocytosis in the TME may be disrupted by SIRPα interactions [113]. CD47 is a signaling receptor overexpressed in cancer cells that triggers the “do not eat me” signal. CD47 binds to SIRPα expressed by TAMs and initiates a signaling cascade to inhibit TAM phagocytosis [114]. Targeting CD47 and blocking the CD47–SIRPα interaction can eliminate this inhibitory signal and enhance macrophage phagocytosis, allowing T cells to fulfill their tumor-killing role in the TME [115]. Huang et al. developed CAR-T cells secreting the CD47-blocking fusion protein SIRPα-Fc to target the CD47-SIRPα pathway in the TME [116]. These armored CAR-T cells not only increased the proportion of M1-type macrophages and improved the phagocytic ability of macrophages, resulting in the remodeling of the immune environment of the TME and reducing immunosuppression in tumor tissues, but also exhibited antitumor effects and persistence in a variety of solid tumors, including breast cancer and colon cancer.

#### Triggering receptor expressed on myeloid cells 2

Triggering receptor expressed on myeloid cells 2 (TREM2) is an immunoglobulin superfamily transmembrane receptor that is predominantly expressed in peripheral macrophages and exerts its function on various immune cells such as TAMs and MDSCs [117]. In a recent study, Huang et al.. generated autocrine PD-1-TREM2 single-chain antibody CAR-T cells by constructing a PD-1-TREM2 bispecific single-chain fragment variant using TREM2 antibody and PD-1 antibody [33]. The continuously secreted PD-1-TREM2 scFv could target the TME, block the PD-1/PD-L1 signaling pathway, as well as ligand binding to the TREM2 receptor present in MDSCs and TAMs, thereby reversing its immunosuppressive effects, and showed superior antitumor efficacy in colorectal cancer.

### Targeting immune checkpoints

Immune checkpoints are immunosuppressive molecules expressed on immune cells that regulate the degree of immune activation and play an important role in regulating immune function [118]. However, some immune checkpoint factors, such as PD-1 and CTLA-4, suppress T cell functions [119]. When bound to their ligands, these immunosuppressive immune checkpoint proteins inhibit the activation of immunoreactive pathways, thereby affecting the activation, proliferation, and survival of T cells [120]. In CAR-T cell therapy, targeting and inhibiting overexpressed immune checkpoints are important to counteract the barriers to T cell function and enhance the role of CAR-T cells in solid tumors (Fig. 4B) [74].

#### CTLA-4

CTLA-4, also known as CD152, is an immune checkpoint molecule. CTLA-4-expressing T cells prevent CTL activation by competing with CD28 for binding to CD80/CD86 molecules on APCs [121]. In addition, Tregs also decrease the CD80/CD86 expression in APCs *via* CTLA-4, further inhibiting T cell effector functions [122]. Hence, targeting this molecule may enhance the therapeutic response of T cells. Ling et al. developed armored CAR-T cells (CTLA-4-CAR-T cells) containing the CD28 structural domain and the extracellular and transmembrane structural domains of CTLA-4, which showed excellent cytokine secretion and antitumor activities, enhancing immune responses in the TME [123]. In further studies, Chen et al. fused the CTLA-4 cytoplasmic tail with the C-terminus of the CAR, leveraging the endocytic properties of the CTLA-4 tail [124]. This approach significantly reduced CAR-mediated phagocytosis, thereby enhancing the antitumor efficacy of CAR-T cells while also achieving sustained therapeutic effects.

#### Targeting the PD-1/PD-L1 axis

PD-1, an immune checkpoint inhibitor, inhibits T-lymphocyte function by binding to PD-L1/PD-L2 ligands, thus mediating T cell apoptosis or inducing T cell exhaustion [125]. This has been reported to inhibit the autoimmune response and limit the immune response in vivo. Rafiq et al. modified CAR-T cells to secrete a scFv blocked by PD-1 to target the TME [74]. In clinically relevant mouse models of PD-L1^+^ solid tumors, CAR-T cells secreted scFvs in a paracrine and autocrine manner, enhancing the antitumor activity of T cells without the toxicity associated with checkpoint inhibition. Professor Qian et al.. announced the first clinical trial of autocrine PD-1 CAR-T cell therapy for ovarian cancer. The results showed that not only the number of CAR-T cells in the blood of the infused patients was expanded, but also the activity of the patient’s own CD8^+^T cells was enhanced, indicating that CAR-T cells infused into the human body mobilize the body’s immune cells to synergistically kill tumor cells, thereby improving antitumor efficacy [73]. Moreover, patients experienced only mild side effects, such as grade 1 hypertension and fatigue, indicating that the CAR-T cells have a favorable safety profile.

#### TIM3

The immune checkpoint TIM3 is a type I membrane protein expressed on the surface of both CD4^+^ and CD8^+^T cell subsets [126]. In the TME, the major role of TIM3 is to inhibit the activation and proliferation of T cells and reduce the production of cytokines that positively regulate the immune response but negatively regulate the antitumor immune response, thus causing the immune escape of tumor cells [127]. Jafarzadeh et al. designed three different shRNA sequences specifically targeting the human TIM3 gene and inserted them into lentiviral vectors together with a MSLN transgene to construct MSLN-CAR-T cells [128]. Accordingly, TIM3 expression in MSLN-CAR-T cells was significantly reduced, whereas cytotoxicity and T cell proliferation were significantly improved following *TIM3* knockdown. This finding demonstrated that TIM3 downregulation could expand the number and promote the effector function of tumor-infiltrating CAR-T cells by attenuating the immunosuppressive effects of TIM3 signaling.

#### TIGIT

T cell immunoglobulin and ITIM domain (TIGIT) is a newly discovered inhibitory immune checkpoint expressed in several immune cells, including CD8^+^T cells, Tregs, and natural killer cells in solid tumors, where it negatively regulates their function [126]. Additionally, TIGIT can inhibit immune cells at multiple steps of the tumor immune cycle, impeding immune responses [129]. Guo et al. used genetic engineering techniques to modify MSLN CAR-T cells to autocrine TIGIT scFv for the treatment of a mouse model established with injected Hela cells. The experimental results showed that targeting and blocking TIGIT could promote cytokine release and enhance the infiltration and activation of CAR-T cells in the TME, thereby enhancing the tumor-killing ability in vivo [130].

#### BTLA-herpes virus entry mediator (HVEM) axis

Studies have shown that the interaction between B- and T-lymphocyte attenuator (BTLA) on effector T cells and HVEM, also known as TNFRSF14) on Tregs within the TME plays a crucial role in establishing immunosuppression [131]. Notably, high levels of BTLA expression in CAR-T cells are associated with poor clinical response, whereas HVEM expression has been recognized as a significant checkpoint in various hematologic and solid malignancies, in some cases even surpassing PD-L1 expression [132]. Based on these insights, researchers have engineered CAR-T cells with deleted BTLA, leading to enhanced tumor control and persistence in models of lymphoma and solid tumors. Mechanistically, BTLA recruits tyrosine phosphatases SHP-1 and SHP-2 through its interaction with HVEM, thereby inhibiting CAR-T cell function. Deletion of BTLA promotes CAR signaling and enhances effector function [133]. Overall, these findings highlight the BTLA-HVEM axis as a crucial immune checkpoint in CAR-T cell immunotherapy, suggesting that strategies to disrupt this pathway may improve therapeutic outcomes.

#### B7H6

In recent years, research on the B7 family of immune checkpoint molecules has increased significantly. Beyond PD-L1 (B7-H1), attention has also been directed to other members of the B7 family, including B7-H3 (CD276), B7-H4, B7-H5 (Vista), B7-H6, and B7-H7 (HHLA2). These molecules can play various roles in tumor progression through interactions with specific ligands [134]. Notably, B7H6 is present in solid tumor cell lines such as melanoma, breast cancer, and pancreatic cancer, where it promotes tumor immune evasion by binding to inhibitory receptors [135]. Building on this knowledge, Gacerez and Sentman engineered a B7H6-specific CAR, integrating it with different variants of T-bet to promote a Th1 phenotype in CD4 + T cells. This strategic modification aims to enhance CAR-T cell effectiveness. Their findings suggest that this modification could alter the TME and activate an immune response, improving the treatment of both solid and hematologic cancers [136]. This highlights the potential of targeting B7 family molecules, particularly B7H6, as a strategy to overcome immune resistance in cancer therapy.

#### Other potential immune checkpoints

Neuronilin-1 (NRP1) has garnered significant attention in the field of immuno-oncology due to its dual functions—enhancing the immunosuppressive activity of Tregs and limiting the sustained response of CD8^+^T cells [136]. NRP1 is crucial for maintaining the function and phenotype of intratumoral Tregs. Disruption of the NRP1 pathway, either through antibody blockade or Treg-specific gene deletion (via Foxp3CreNrp1L/L), can impair the suppressive function of intratumoral Tregs, thereby restoring antitumor immunity [137]. Furthermore, NRP1 intrinsically affects the development and function of intratumoral CD8^+^T cells and selectively influences the generation of memory precursors during antitumor immune responses. A combined blockade of PD-1 and NRP1 has been shown to result in more durable systemic antitumor immunity and long-term remission in cancer patients [138]. Additionally, NR2F6, also known as Ear-2 or COUP-TFIII, a member of the NR2F subfamily, is upregulated in various human solid tumors and has been identified as an immune checkpoint central to the suppression of antitumor responses [139]. Mechanistically, NR2F6 expressed in lymphocytes acts as a key regulator during T-lymphocyte activation, fine-tuning adaptive immunity and inhibiting the transcription of genes encoding cytokines such as IL-2, IFN-γ, IL-17, and IL-21. In genetically engineered NR2F6-deficient mouse models, the absence of NR2F6 leads to enhanced activity of tumor-infiltrating effector T cells, resulting in increased secretion of IFN-γ and IL-2 and augmented memory function of CD8^+^T cells, demonstrating superior efficacy compared to PD-1 monotherapy [140]. Moreover, inhibiting NR2F6 also enhances the response to PD-1 therapy. Preclinical studies have further demonstrated the feasibility of combining NRP1 and NR2F6 antagonism with established immunotherapies [138]. Looking ahead, exploring the combination of emerging immune checkpoints with CAR-T cell therapy holds great promise. This approach could potentially overcome resistance and tumor evasion mechanisms inherent in current treatments, thereby enhancing the antitumor activity of CAR-T cells and improving the durability and breadth of therapeutic responses.

### Targeting DCs

DCs are a highly heterogeneous group of specialized APCs with a great capacity to take up, process, and present antigens [141]. Conventional DCs (cDCs) are subdivided into two subpopulations with different functional characteristics that exert distinct immunostimulatory and immunosuppressive effects in tumor tissues and organs: cDC1 expressing CD141 and cDC2 expressing CD1c [142]. cDC1 induces a potent antitumor CTL response, whereas cDC2 induces an Th17 cell-based immune response, thereby enhancing the therapeutic promise of CAR-T cells that promote DC proliferation in the TME of solid tumors (Fig. 4B) [143].

#### FMS-like tyrosine kinase 3 ligand

FMS-like tyrosine kinase 3 ligand (FLT3L) is associated with DC proliferation [144]. Binding of FLT3L to its receptor FLT3, specifically in the TME, induces DC expansion in circulation, thereby promoting their immune function and tumor-killing behavior. Beavis et al.. designed CAR-T cells that secrete FLT3L, successfully promoting the infiltration of cDC1 and CD8^+^T cells into the TME of sarcoma and colorectal adenocarcinoma and significantly enhancing the activation of host DCs and T cells. More importantly, the combination of CAR-T cells with TCR-T cells further inhibited tumor growth and, through antigen spreading, induced immune responses against epitopes beyond the recognition scope of the adoptively transferred T cells [145]. This study suggests that increasing the number of endogenous DCs is a promising strategy to overcome the challenge of antigen-negative tumor escape following adoptive cell therapy.

#### CD40

CD40, a member of the TNF receptor superfamily, is expressed on a variety of APCs, including DCs, and is essential for their activation and proliferation [146]. It primarily interacts with the CD40 ligand (CD40L) expressed by CD4^+^T cells to regulate T cell-dependent antitumor immunity. Activation of the CD40-CD40L axis upregulates MHC and produces pro-inflammatory cytokines such as IL12, which are essential for T cell initiation [147]. Brentjens et al. engineered a tumor-targeting CAR-T cell modified with CD40L, which achieved promising antitumor effects by inducing an endogenous antitumor response in several models of both leukemia and lymphoma [148]. In the field of solid tumors, Zhang et al.. engineered a secretory anti-CD40 antibody with the ability to generate MSLN3-CD40 CAR-T cells targeting the MSLN III region, which showed potential antitumor activity by activating the CD40 pathway [149]. Compared to CAR-T cells that did not secrete anti-CD40 antibodies, MSLN3-CD40 CAR-T cells secreted increased levels of cytokines in response to target antigen stimulation and enhanced antitumor activity in a xenograft tumor model established by subcutaneous inoculation of 1 × 10^7^ SKOV-3-luc cells.

### Targeting the vascular system

Compared to healthy tissue, the tumor vasculature often exhibits multiple morphological, functional, and metabolic abnormalities due to high EC rates and abnormal pericyte coverage [150]. This abnormal vasculature also leads to insufficient oxygen delivery to the TME, exacerbating hypoxia within the tumor and increasing tumor invasiveness [151]. Moreover, dysfunctional blood vessels prevent the function of immune cells in the TME, not only by preventing T cell infiltration but also by interfering with the transport and delivery of therapeutic agents [152]. Given the inhibitory functions of the vasculature in the TME, modifying CAR-T cells to target the vasculature is important to improve their application in solid tumors (Fig. 4B) [153].

#### Vascular endothelial growth factor

Vascular endothelial growth factor (VEGF), which is secreted by tumor and stromal cells, exerts multiple functions in the TME [154]. In particular, it not only stimulates the proliferation of ECs, leading to neointimal formation and increased vascular permeability, but also leads to transient fibrin matrix deposition, triggering connective tissue mesenchymal formation [155]. In addition, VEGF has an autocrine function that promotes dedifferentiation and epithelial-mesenchymal transition, thereby enhancing tumor invasion and survival. Inhibiting the expression of VEGF and VEGFR and blocking the tumor cell signal transduction pathway inhibits neoangiogenesis and suppresses tumor growth and metastasis [156]. Wei et al.. prepared a human VEGFR-1-specific CAR (V-1 CAR) capable of targeting VEGFR and used it to generate a novel CAR-T cell. These CAR-T cells successfully delayed tumor growth and formation and effectively inhibited tumor metastasis in a xenograft lung tumor model established by subcutaneous inoculation of A549 cells into NOD-SCID BALB/c mice [157]. In addition, the researchers found that these newly developed V-1 CAR-T cells blocked blood vessel formation by lysing ECs in vitro. It may be possible to use them in combination with conventional anti-vascular therapy for the treatment of solid tumors. However, since VEGFR is not only expressed on cancer cells, such CAR-modified T lymphocytes must avoid contact with antigens in normal tissues to prevent severe side effects. In addition, since the expression of CAR in such CAR-T cells transiently modified by non-viral vectors gradually declines as they kill tumors, their expression level in vivo must be maintained by continuous supplemental infusion. In a follow-up study, Wang et al.. developed a T cell expressing both VEGFR2- and VEGFR3-specific chimeric antigens for CAR-T therapy, which showed promising antitumor effects in both VEGFR-2- and VEGFR-3-positive breast cancers and significantly inhibited in situ mammary xenograft tumor growth, infiltration, and metastasis in female BALB/c nude mice [158]. These findings suggest that vascular-targeted therapy against VEGF has the potential to be used in conjunction with conventional anti-angiogenic therapies and provide theoretical support for the clinical translation of modified vascular-targeted CAR-T cells.

#### Fibroblast growth factor receptor

Fibroblast growth factor (FGF) is a mammalian protein that promotes cell division. It stimulates the growth and differentiation of various cells and aids in small blood vessel regeneration [159]. Epidermal growth factor receptor-mediated pathways are crucial for normal cell growth and differentiation and support new blood vessel formation [160]. However, mutations or overexpression of FGFR may lead to excessive activation of the FGFR signaling pathway, potentially leading to carcinogenesis. Hence, targeting the FGFR pathway may help prevent carcinogenesis and the formation of an abnormal vasculature in tumors [161].

Based on the specific anti-FGFR4 antibody 3A11, Tian et al.. designed a FGFR4-targeting CAR as a novel T cell therapy for rhabdomyosarcoma (RMS) [162]. They reported that such 3A11-CAR-T cells induced the secretion of high levels of cytokines in RMS cells, producing potent cytotoxicity in vitro. In addition, these CAR-T cells exhibited good persistence in vivo and effectively eliminated RMS tumors in two metastatic and two orthotopic models, providing the promising application of FGFR4-targeting CAR-T cell therapy in patients with RMS.

### Targeting the ECM and CAFs

The ECM is a network of fibrous components that participate in intercellular information transfer while regulating cell behavior and immune responses [163]. Abnormal accumulation of ECM molecules in the TME creates a physical barrier that hinders drug therapy [164]. CAFs are key producers of ECM molecules in the TME. They support tumor growth by depositing ECM molecules and producing enzymes that remodel the stroma, thereby promoting tumor spread and metastasis [66]. In addition, CAFs secrete cytokines, exosomes, and growth factors that promote tumor growth and invasion [165]. Consequently, one approach for improving the efficacy of armored CAR-T cells is to directly target ECM and CAFs in the TME, disrupting tumor growth and resistance while improving treatment outcomes (Fig. 4B).

#### Fibroblast activation protein

Fibroblast activation protein (FAP) is overexpressed in a subpopulation of CAFs and is highly expressed in more than 90% of solid tumors, making it a suitable target for CAR-T cell therapy [166]. Ellen Puré et al.. designed FAP-CAR-T cells capable of targeting FAP in the TME [167]. Using several pancreatic cancer mouse models, they confirmed that these cells infiltrated tumors rapidly, overcame immune rejection and immunosuppression in the TME, and exerted significant antitumor effects.

#### TGF-β

The TGF-β signaling pathway may either promote or inhibit tumor growth [168]. TGF-β primarily influences the TME, where it suppresses T cells, macrophages, and neutrophils, weakens the ability of the host to counter tumor development, and helps tumor cells in evading the immune system [169]. Hence, TGF-β has been suggested as a new target for CAR-T cell therapy. Carl’s team overexpressed a dominant-negative mutant of the TGF-β R II gene in CAR-T cells targeting PSMA, which significantly enhanced the ability of CAR-T cells to infiltrate, proliferate, and mediate antitumor responses in a prostate cancer model by inhibiting the TGF-β signaling pathway within tumors [70]. They have recently announced a Phase I clinical trial to evaluate this new approach to CAR-T cells as a treatment for recurrent and refractory metastatic prostate cancer [82]. This clinical trial demonstrated the feasibility and safety of these CAR-T cells, evaluating their distribution in vivo, bioactivity, and disease response.

#### CXCR 4/5

CXCR4 and its ligands have been linked to tumor immunity, cancer development, and metastasis [76]. Hence, targeting this molecule may be beneficial in cancer treatment. Chemokines that bind to these receptors affect the movement, maturation, and development of immune responses. Blocking CXCR4 can alter tumor-environment interactions, increase cancer cell susceptibility to drugs, and reduce tumor growth and migration [170]. Li et al.. constructed CLDN18.2-targeting CAR-T cells co-expressing CXCR4 and showed that CXCR4-CAR-T cells were better at infiltrating the tumor and inhibiting MDSC migration through the STAT3/NF-κB/SDF-1α axis, resulting in improved efficacy against CLDN18.2-positive pancreatic cancer. These findings provide a theoretical basis for the construction of CXCR-CAR-T cells for the treatment of solid tumors [171].

Currently, as more and more of the above strategies have been shown to be effective in improving the efficacy of armored CAR-T in solid tumors in preclinical studies, their translatability to clinical applications is receiving widespread attention. Several clinical trials of armored CAR-T navigating solid tumors are underway, gradually exploring the translational potential from preclinical studies to clinical applications. (Table 1).

**Table 1:** Summary of current clinical trials on armored CAR-T therapy for human solid tumors

Identifier	Armor	Target antigen	Tumor type(s)	Clinical stage	Primary outcome (s)
NCT04556669	Carry a scFv fragment of anti-PD-L1 monoclonal antibody	CD22	Cervical Cancer/ NSCLC/Sarcoma	I	AEs
NCT06084286	Construct CLDN18.2/PD-L1 dual-targeting CAR-T	Claudin18.2	CLDN18.2-positive Solid Tumors	I	AEs/DLT/RP2D
NCT02873390	Engineer T cells to express PD-1 antibodies	EGFR	EGFR-positive Solid Tumors	I/II	ORR/DCR/OS/PFS
NCT02862028	Engineer T cells to express PD-1 antibodies	EGFR	EGFR-positive Lung and Stomach Cancers	I/II	ORR/DCR/OS/PFS
NCT03182816	Engineer T cells to express PD-1 and CTLA-4 antibodies	EGFR	MSLN-positive Solid Tumors	I/II	AEs
NCT05060796	Modify EGFR CAR-T with CXCR5	EGFR	EGFR-positive NSCLC	I	AEs
NCT04153799	Modify EGFR CAR-T with CXCR5	EGFR	EGFR-positive NSCLC	I	AEs /ORR
NCT03198546	Construct CAR-T cells that target GPC3 and soluble TGF-β	GPC3	HCC	I	DLT
NCT05155189	TGFβRIIDN armored autologous CAR-T	GPC3	HCC	I	TEAEs/AESIs
NCT03198052	Engineer T cells to express TGF-β-CAR and secrete scfv against PD-1 /CTLA4/TIGIT	GPC3/HER2 EGFR/MSLN	Lung Cancer	I	DLT
NCT06249256	Secrete PD-1 nanobodies	MSLN	MSLN-positive Solid Tumors	I	DLT
NCT05779917	Secrete a fusion protein of IL-21 and scfv against PD-1	MSLN	Pancreas Cancer	I	DLT
NCT05373147	Secrete PD-1 nanobodies	MSLN	MSLN-positive Solid Tumors	I	DLT
NCT05089266	Secrete PD-1 nanobodies	MSLN	Colorectal Cancer	I	DLT
NCT04577326	Link anti-MSLN scFv to the CD28 and CD3ζ	MSLN	Malignant Pleural Mesothelioma	I	AEs
NCT06248697	Secrete PD-1/CTLA-4 nanobodies	MSLN	NSCLC	I	DLT
NCT04489862	Secrete PD-1 nanobodies	MSLN	NSCLC	I	DLT
NCT03615313	Engineer T cells to express PD-1 antibodies	MSLN	MSLN-positive Solid Tumors	I/II	TRAE
NCT03030001	Engineer T cells to express PD-1 antibodies	MSLN	MSLN-positive Solid Tumors	I/II	AEs
NCT03182803	Engineer T cells to express PD-1 and CTLA-4 antibodies	MSLN	MSLN-positive Solid Tumors	I/II	AEs
NCT04503980	Secrete PD-1 nanobodies	MSLN	Colorectal Cancer/Ovarian Cancer	I	DLT
NCT05944185	Secrete PD-1 nanobodies	MSLN	MSLN-positive Solid Tumors	I/II	AEs /MTD/ORR
NCT03179007	Engineer T cells to express PD-1 and CTLA-4 antibodies	MUC1	MUC1-positive Solid Tumors	I/II	AEs
NCT01722149	Engineer CAR-T to express IL-7 and CCL19, or IL12 to target FAP	Nectin4	Nectin4-positive Solid Tumor	I	AEs
NCT04768608	Integrate Anti-PSMA-CAR-T with Non-viral PD-1	PSMA	Castrate-Resistant Prostate Cancer	I	AEs
NCT06046040	Dually armored CAR-T with a TGFβRDN and PD-1	PSMA	Metastatic Castrate-Resistant Prostate Cancer	I	DLTs/MTD/AEs
NCT03089203	Modify CAR-T to construct TGFβ-resistant CAR-T cells	PSMA	Prostate Cancer	I	AEs
NCT05477927	Dual-targeting VEGFR1 and PD-L1 CAR-T	VEGFR1	Pleural or Peritoneal Metastatic Carcinoma	I	AEs /DLT

*Abbreviations* AEs (Adverse Events), AESIs (Adverse Events of Special Interest), DCR (Disease Control Rate), DLT (Dose-Limiting Toxicity), HCC (Hepatocellular Carcinoma), MTD (Maximum Tolerated Dose), NSCLC (Non-Small Cell Lung Cancer), ORR (Objective Response Rate), OS (Overall survival), PFS (Progression-Free Survival), RP2D (Recommended Phase II Dose), TEAEs (Treatment-Emergent Adverse Events), and TRAE (Treatment-Related Adverse Event)

## Conclusions

CAR-T cell therapy is successful against hematologic tumors but presents challenges in treating solid tumors, primarily owing to suboptimal efficacy in the immunosuppressive TME of tumors. However, novel enhanced armored CAR-T cells are being developed to overcome these challenges [172]. Research has identified antigenic modulation and T cell dysfunction as key mechanisms of tumor resistance, suggesting potential avenues to enhance the efficacy of CAR-T cell therapy against solid tumors. Next-generation CAR-T cells are likely to be equipped with specific factors designed to overcome various barriers, navigate the TME, and deliver bioactive molecules to counteract its protective effects on tumors. Given the complexity of the TME, identifying additional target molecules responsible for treatment resistance across different cancers is crucial for further improving therapeutic outcomes [6].

Future studies should aim to elucidate the composition of the TME and develop strategies to counteract its immunosuppressive mechanisms while activating antitumor immunity, which may help to improve the efficacy of immunotherapies. In addition to the current therapeutic targets used in the design of the armored CAR-T cells mentioned in this review, several potential targets in the TME remain to be uncovered and warrant further studies. Over the past decade, most approaches have utilized specific cytokines or other functional proteins, including antibody proteins, to armor CAR-T cells. In the future, the tools for armoring CAR-T cells may be extended to a wider range of bioactive substances, including DNA, non-coding RNAs, and functional peptides, thus increasing the possibility of navigating the TME [72]. Furthermore, future CAR-T cells could be armored with a series of molecular components that work together to form a therapeutic biological microsystem, thereby realizing intelligent cancer treatment by CAR-T cells [172].

In summary, based on our understanding of the complexity of the TME composition and the discovery of special therapeutic targets, novel CAR-T cells navigating the TME are expected to efficiently target and kill tumors [16]. This novel strategy of armoring CAR-T cells with therapeutic targets may improve their efficacy against solid tumors, achieving important clinical application value.

## Data Availability

No datasets were generated or analysed during the current study.

## Abbreviations

ADCC: Antibody-dependent cell-mediated cytotoxicity
APC: Antigen-presenting cell
BTLA: B- and T-lymphocyte attenuator
CAF: Cancer-associated fibroblast
CAR: Chimeric antigen receptor
CCL: C-C chemokine ligand
CCR: C-C chemokine receptor
cDC: Classical dendritic cell
CEA: Carcinoembryonic antigen
CSF1R: Colony-stimulating factor 1 receptor
CTLA-4: Cytotoxic T-lymphocyte-associated antigen 4
CXCR: C-X-C chemokine receptor
DC: Dendritic cell
EC: Endothelial cell
ECM: Extracellular matrix
FAP: Fibroblast activation protein
FGF: Fibroblast growth factor
FLT3L: FMS-like tyrosine kinase 3 ligand
GPC3: Glypican-3
HEVM: Herpes virus entry mediator
HPSE: Heparanase
IDO: Indoleamine 2,3-dioxygenase
IFN-γ: Interferon-γ
IL: Interleukin
LAG-3: Lymphocyte-activation gene 3
MDSC: Bone marrow-derived suppressor cell
MHC: Major histocompatibility complex
MMP: Matrix metalloproteinase
MSLN: Mesothelin
NK: Natural killer cell
NRP1: Neuronilin-1
OTOT: On-target/offtumor toxicity
PD-1: Programmed cell death protein 1
pDC: Plasmacytoid dendritic cell
RMS: Rhabdomyosarcoma
ROS: Reactive oxygen species
scFv: Single-chain variable fragment
SIRPα: Signaling regulatory proteinα
TAM: Tumor-associated macrophage
TGF-β: Transforming growth factor beta
TIGIT: T cell immunoglobulin and ITIM domain
TIM3: T cell immunoglobulin domain and mucin domain 3
TME: Tumor microenvironment
Treg: Regulatory T cell
TREM2: Triggering receptor expressed on myeloid cells 2
TRUCK: T cells redirected to universal cytokine killing
VEGF: Vascular endothelial growth factor
V_H_: Variable regions of heavy chain
V_L_: Variable regions of light chain

## References

[1] Albelda SM. CAR T cell therapy for patients with solid tumours: key lessons to learn and unlearn. Nat Rev Clin Oncol. 2024;21(1):47–66. 37904019 10.1038/s41571-023-00832-4

[2] Mullard A. FDA approves fourth CAR-T cell therapy. Nat Rev Drug Discov. 2021;20(3):166. 10.1038/d41573-021-00031-9 33574586

[3] Sterner RC, Sterner RM. CAR-T cell therapy: current limitations and potential strategies. Blood Cancer J. 2021;11(4):69. 33824268 10.1038/s41408-021-00459-7 PMC8024391

[4] Maalej KM, Merhi M, Inchakalody VP, Mestiri S, Alam M, Maccalli C, et al. CAR-cell therapy in the era of solid tumor treatment: current challenges and emerging therapeutic advances. Mol Cancer. 2023;22(1):20. 36717905 10.1186/s12943-023-01723-z PMC9885707

[5] Qu C, Zhang H, Cao H, Tang L, Mo H, Liu F, et al. Tumor buster - where will the CAR-T cell therapy ‘missile’ go? Mol Cancer. 2022;21(1):201. 36261831 10.1186/s12943-022-01669-8 PMC9580202

[6] de Visser KE, Joyce JA. The evolving tumor microenvironment: from cancer initiation to metastatic outgrowth. Cancer Cell. 2023;41(3):374–403. 36917948 10.1016/j.ccell.2023.02.016

[7] Shi Y, Shi D, Chi J, Cui D, Tang X, Lin Y, et al. Combined local therapy and CAR-GPC3 T-cell therapy in advanced hepatocellular carcinoma: a proof-of-concept treatment strategy. Cancer Commun. 2023;43(9):1064–68. 10.1002/cac2.12472 PMC10508142 37478283

[8] Zhao Y, Chen J, Andreatta M, Feng B, Xie YQ, Wenes M et al. IL-10-expressing CAR T cells resist dysfunction and mediate durable clearance of solid tumors and metastases. Nat Biotechnol. 2024. 10.1038/s41587-023-02060-8 38168996

[9] Larson RC, Maus MV. Recent advances and discoveries in the mechanisms and functions of CAR T cells. Nat Rev Cancer. 2021;21(3):145–61. 33483715 10.1038/s41568-020-00323-z PMC8353572

[10] Safarzadeh Kozani P, Naseri A, Mirarefin SMJ, Salem F, Nikbakht M, Evazi Bakhshi S, et al. Nanobody-based CAR-T cells for cancer immunotherapy. Biomark Res. 2022;10(1):24. 35468841 10.1186/s40364-022-00371-7 PMC9036779

[11] Cappell KM, Kochenderfer JN. A comparison of chimeric antigen receptors containing CD28 versus 4-1BB costimulatory domains. Nat Rev Clin Oncol. 2021;18(11):715–27. 34230645 10.1038/s41571-021-00530-z

[12] Tousley AM, Rotiroti MC, Labanieh L, Rysavy LW, Kim WJ, Lareau C, et al. Co-opting signalling molecules enables logic-gated control of CAR T cells. Nature. 2023;615(7952):507–16. 36890224 10.1038/s41586-023-05778-2 PMC10564584

[13] Lu J, Jiang G. The journey of CAR-T therapy in hematological malignancies. Mol Cancer. 2022;21(1):194. 36209106 10.1186/s12943-022-01663-0 PMC9547409

[14] Abreu TR, Fonseca NA, Gonçalves N, Moreira JN. Current challenges and emerging opportunities of CAR-T cell therapies. J Control Release. 2020; 319(246 – 61. 10.1016/j.jconrel.2019.12.047 31899268

[15] Liu J, Jiao X, Ma D, Fang Y, Gao Q. CAR-T therapy and targeted treatments: emerging combination strategies in solid tumors. Med. 2024;5(6):530–49. 38547867 10.1016/j.medj.2024.03.001

[16] Hou AJ, Chen LC, Chen YY. Navigating CAR-T cells through the solid-tumour microenvironment. Nat Rev Drug Discov. 2021;20(7):531–50. 33972771 10.1038/s41573-021-00189-2

[17] Ma L, Hostetler A, Morgan DM, Maiorino L, Sulkaj I, Whittaker CA, et al. Vaccine-boosted CAR T crosstalk with host immunity to reject tumors with antigen heterogeneity. Cell. 2023;186(15):3148–e6520. 37413990 10.1016/j.cell.2023.06.002 PMC10372881

[18] Flugel CL, Majzner RG, Krenciute G, Dotti G, Riddell SR, Wagner DL, et al. Overcoming on-target, off-tumour toxicity of CAR T cell therapy for solid tumours. Nat Rev Clin Oncol. 2023;20(1):49–62. 36418477 10.1038/s41571-022-00704-3 PMC10278599

[19] Thistlethwaite FC, Gilham DE, Guest RD, Rothwell DG, Pillai M, Burt DJ, et al. The clinical efficacy of first-generation carcinoembryonic antigen (CEACAM5)-specific CAR T cells is limited by poor persistence and transient pre-conditioning-dependent respiratory toxicity. Cancer Immunol Immunother. 2017;66(11):1425–36. 28660319 10.1007/s00262-017-2034-7 PMC5645435

[20] Xie N, Shen G, Gao W, Huang Z, Huang C, Fu L. Neoantigens: promising targets for cancer therapy. Signal Transduct Target Ther. 2023;8(1):9. 36604431 10.1038/s41392-022-01270-x PMC9816309

[21] Linnemann C, van Buuren MM, Bies L, Verdegaal EM, Schotte R, Calis JJ, et al. High-throughput epitope discovery reveals frequent recognition of neo-antigens by CD4 + T cells in human melanoma. Nat Med. 2015;21(1):81–5. 25531942 10.1038/nm.3773

[22] Gumber D, Wang LD. Improving CAR-T immunotherapy: Overcoming the challenges of T cell exhaustion. EBioMedicine. 2022; 77(103941. 10.1016/j.ebiom.2022.103941 PMC8927848 35301179

[23] ElTanbouly MA, Noelle RJ. Rethinking peripheral T cell tolerance: checkpoints across a T cell’s journey. Nat Rev Immunol. 2021;21(4):257–67. 33077935 10.1038/s41577-020-00454-2 PMC12536352

[24] Propper DJ, Balkwill FR. Harnessing cytokines and chemokines for cancer therapy. Nat Rev Clin Oncol. 2022;19(4):237–53. 34997230 10.1038/s41571-021-00588-9

[25] Kenison JE, Stevens NA, Quintana FJ. Therapeutic induction of antigen-specific immune tolerance. Nat Rev Immunol. 2024;24(5):338–57. 38086932 10.1038/s41577-023-00970-x PMC11145724

[26] Delgoffe GM, Xu C, Mackall CL, Green MR, Gottschalk S, Speiser DE, et al. The role of exhaustion in CAR T cell therapy. Cancer Cell. 2021;39(7):885–88. 34256903 10.1016/j.ccell.2021.06.012

[27] Peng JJ, Wang L, Li Z, Ku CL, Ho PC. Metabolic challenges and interventions in CAR T cell therapy. Sci Immunol. 2023;8(82):eabq3016. 37058548 10.1126/sciimmunol.abq3016

[28] Van der Vreken A, Vanderkerken K, De Bruyne E, De Veirman K, Breckpot K, Menu E. Fueling CARs: metabolic strategies to enhance CAR T-cell therapy. Exp Hematol Oncol. 2024;13(1):66. 38987856 10.1186/s40164-024-00535-1 PMC11238373

[29] Caruana I, Savoldo B, Hoyos V, Weber G, Liu H, Kim ES, et al. Heparanase promotes tumor infiltration and antitumor activity of CAR-redirected T lymphocytes. Nat Med. 2015;21(5):524–9. 25849134 10.1038/nm.3833 PMC4425589

[30] Gunderson AJ, Yamazaki T, McCarty K, Fox N, Phillips M, Alice A, et al. TGFβ suppresses CD8(+) T cell expression of CXCR3 and tumor trafficking. Nat Commun. 2020;11(1):1749. 32273499 10.1038/s41467-020-15404-8 PMC7145847

[31] Rodriguez-Garcia A, Palazon A, Noguera-Ortega E, Powell DJ Jr., Guedan S. CAR-T Cells Hit the Tumor Microenvironment: Strategies to Overcome Tumor Escape. Front Immunol. 2020; 11(1109. 10.3389/fimmu.2020.01109 PMC7311654 32625204

[32] Sterner RM, Sakemura R, Cox MJ, Yang N, Khadka RH, Forsman CL, et al. GM-CSF inhibition reduces cytokine release syndrome and neuroinflammation but enhances CAR-T cell function in xenografts. Blood. 2019;133(7):697–709. 30463995 10.1182/blood-2018-10-881722 PMC6376281

[33] Chen J, Zhu T, Jiang G, Zeng Q, Li Z, Huang X. Target delivery of a PD-1-TREM2 scFv by CAR-T cells enhances anti-tumor efficacy in colorectal cancer. Mol Cancer. 2023;22(1):131. 37563723 10.1186/s12943-023-01830-x PMC10413520

[34] Togashi Y, Shitara K, Nishikawa H. Regulatory T cells in cancer immunosuppression - implications for anticancer therapy. Nat Rev Clin Oncol. 2019;16(6):356–71. 30705439 10.1038/s41571-019-0175-7

[35] Sun R, Luo H, Su J, Di S, Zhou M, Shi B, et al. Olaparib suppresses MDSC Recruitment via SDF1α/CXCR4 Axis to improve the anti-tumor efficacy of CAR-T cells on breast Cancer in mice. Mol Ther. 2021;29(1):60–74. 33010818 10.1016/j.ymthe.2020.09.034 PMC7791086

[36] Vander Heiden MG, Cantley LC, Thompson CB. Understanding the Warburg effect: the metabolic requirements of cell proliferation. Science. 2009;324(5930):1029–33. 19460998 10.1126/science.1160809 PMC2849637

[37] Brand A, Singer K, Koehl GE, Kolitzus M, Schoenhammer G, Thiel A, et al. LDHA-Associated Lactic Acid Production blunts Tumor Immunosurveillance by T and NK Cells. Cell Metab. 2016;24(5):657–71. 27641098 10.1016/j.cmet.2016.08.011

[38] Colegio OR, Chu NQ, Szabo AL, Chu T, Rhebergen AM, Jairam V, et al. Functional polarization of tumour-associated macrophages by tumour-derived lactic acid. Nature. 2014;513(7519):559–63. 25043024 10.1038/nature13490 PMC4301845

[39] Rafiq S, Hackett CS, Brentjens RJ. Engineering strategies to overcome the current roadblocks in CAR T cell therapy. Nat Rev Clin Oncol. 2020;17(3):147–67. 31848460 10.1038/s41571-019-0297-y PMC7223338

[40] Srivastava S, Salter AI, Liggitt D, Yechan-Gunja S, Sarvothama M, Cooper K, et al. Logic-gated ROR1 Chimeric Antigen Receptor Expression Rescues T cell-mediated toxicity to normal tissues and enables selective Tumor Targeting. Cancer Cell. 2019;35(3):489–e5038. 30889382 10.1016/j.ccell.2019.02.003 PMC6450658

[41] Li X, Zhu T, Wang R, Chen J, Tang L, Huo W, et al. Genetically programmable vesicles for enhancing CAR-T therapy against solid tumors. Adv Mater. 2023;35(19):e2211138. 36814099 10.1002/adma.202211138

[42] Hu X, Zhang J, Wang J, Fu J, Li T, Zheng X, et al. Landscape of B cell immunity and related immune evasion in human cancers. Nat Genet. 2019;51(3):560–67. 30742113 10.1038/s41588-018-0339-x PMC6773274

[43] Güç E, Pollard JW. Redefining macrophage and neutrophil biology in the metastatic cascade. Immunity. 2021;54(5):885–902. 33979586 10.1016/j.immuni.2021.03.022

[44] Hussein A, Stamova S, Xydia M, Beckhove P. Hand in hand to successful immunotherapy: CD8(+) T cells and M1-like macrophages swap the baton. Cancer Cell. 2024;42(6):938–41. 38861930 10.1016/j.ccell.2024.05.012

[45] Mantovani A, Marchesi F, Malesci A, Laghi L, Allavena P. Tumour-associated macrophages as treatment targets in oncology. Nat Rev Clin Oncol. 2017;14(7):399–416. 28117416 10.1038/nrclinonc.2016.217 PMC5480600

[46] Gerhard GM, Bill R, Messemaker M, Klein AM, Pittet MJ. Tumor-infiltrating dendritic cell states are conserved across solid human cancers. J Exp Med. 2021; 218(1). 10.1084/jem.20200264 PMC7754678 33601412

[47] Heras-Murillo I, Adán-Barrientos I, Galán M, Wculek SK, Sancho D. Dendritic cells as orchestrators of anticancer immunity and immunotherapy. Nat Rev Clin Oncol. 2024. 10.1038/s41571-024-00859-1 38326563

[48] Huang X, Nepovimova E, Adam V, Sivak L, Heger Z, Valko M, et al. Neutrophils in Cancer immunotherapy: friends or foes? Mol Cancer. 2024;23(1):107. 38760815 10.1186/s12943-024-02004-z PMC11102125

[49] Zhang H, Yang L, Wang T, Li Z. NK cell-based tumor immunotherapy. Bioact Mater. 2024;31:63–86. 37601277 10.1016/j.bioactmat.2023.08.001 PMC10432724

[50] Ugel S, Canè S, De Sanctis F, Bronte V. Monocytes in the Tumor Microenvironment. Annu Rev Pathol. 2021;16:93–122. 33497262 10.1146/annurev-pathmechdis-012418-013058

[51] Li K, Shi H, Zhang B, Ou X, Ma Q, Chen Y, et al. Myeloid-derived suppressor cells as immunosuppressive regulators and therapeutic targets in cancer. Signal Transduct Target Ther. 2021;6(1):362. 34620838 10.1038/s41392-021-00670-9 PMC8497485

[52] De Palma M, Biziato D, Petrova TV. Microenvironmental regulation of tumour angiogenesis. Nat Rev Cancer. 2017;17(8):457–74. 28706266 10.1038/nrc.2017.51

[53] Palikuqi B, Nguyen DT, Li G, Schreiner R, Pellegata AF, Liu Y, et al. Adaptable haemodynamic endothelial cells for organogenesis and tumorigenesis. Nature. 2020;585(7825):426–32. 32908310 10.1038/s41586-020-2712-z PMC7480005

[54] Sun R, Kong X, Qiu X, Huang C, Wong PP. The Emerging Roles of Pericytes in Modulating Tumor Microenvironment. Front Cell Dev Biol. 2021; 9(676342. 10.3389/fcell.2021.676342 PMC8232225 34179005

[55] Wei X, Chen Y, Jiang X, Peng M, Liu Y, Mo Y, et al. Mechanisms of vasculogenic mimicry in hypoxic tumor microenvironments. Mol Cancer. 2021;20(1):7. 33397409 10.1186/s12943-020-01288-1 PMC7784348

[56] Li C, Teixeira AF, Zhu HJ, Ten Dijke P. Cancer associated-fibroblast-derived exosomes in cancer progression. Mol Cancer. 2021;20(1):154. 34852849 10.1186/s12943-021-01463-y PMC8638446

[57] Timaner M, Tsai KK, Shaked Y. The multifaceted role of mesenchymal stem cells in cancer. Semin Cancer Biol. 2020; 60(225 – 37. 10.1016/j.semcancer.2019.06.003 31212021

[58] Haj-Shomaly J, Vorontsova A, Barenholz-Cohen T, Levi-Galibov O, Devarasetty M, Timaner M, et al. T cells promote metastasis by regulating Extracellular Matrix Remodeling following Chemotherapy. Cancer Res. 2022;82(2):278–91. 34666995 10.1158/0008-5472.CAN-21-1012 PMC7612244

[59] Yuan Z, Li Y, Zhang S, Wang X, Dou H, Yu X, et al. Extracellular matrix remodeling in tumor progression and immune escape: from mechanisms to treatments. Mol Cancer. 2023;22(1):48. 36906534 10.1186/s12943-023-01744-8 PMC10007858

[60] Huang J, Zhang L, Wan D, Zhou L, Zheng S, Lin S, et al. Extracellular matrix and its therapeutic potential for cancer treatment. Signal Transduct Target Ther. 2021;6(1):153. 33888679 10.1038/s41392-021-00544-0 PMC8062524

[61] Sahai E, Astsaturov I, Cukierman E, DeNardo DG, Egeblad M, Evans RM, et al. A framework for advancing our understanding of cancer-associated fibroblasts. Nat Rev Cancer. 2020;20(3):174–86. 31980749 10.1038/s41568-019-0238-1 PMC7046529

[62] Chen Y, McAndrews KM, Kalluri R. Clinical and therapeutic relevance of cancer-associated fibroblasts. Nat Rev Clin Oncol. 2021;18(12):792–804. 34489603 10.1038/s41571-021-00546-5 PMC8791784

[63] Rimal R, Desai P, Daware R, Hosseinnejad A, Prakash J, Lammers T et al. Cancer-associated fibroblasts: Origin, function, imaging, and therapeutic targeting. Adv Drug Deliv Rev. 2022; 189(114504. 10.1016/j.addr.2022.114504 35998825

[64] Kalluri R. The biology and function of fibroblasts in cancer. Nat Rev Cancer. 2016;16(9):582–98. 27550820 10.1038/nrc.2016.73

[65] Hawkins ER, D’Souza RR, Klampatsa A. Armored CAR T-Cells: the next chapter in T-Cell Cancer immunotherapy. Biologics. 2021;15:95–105. 33883875 10.2147/BTT.S291768 PMC8053711

[66] Posey AD Jr., Young RM, June CH. Future perspectives on engineered T cells for cancer. Trends Cancer. 2024. 10.1016/j.trecan.2024.05.007 38853073

[67] Adachi K, Kano Y, Nagai T, Okuyama N, Sakoda Y, Tamada K. IL-7 and CCL19 expression in CAR-T cells improves immune cell infiltration and CAR-T cell survival in the tumor. Nat Biotechnol. 2018;36(4):346–51. 29505028 10.1038/nbt.4086

[68] Bell M, Lange S, Sejdiu BI, Ibanez J, Shi H, Sun X et al. Modular chimeric cytokine receptors with leucine zippers enhance the antitumour activity of CAR T cells via JAK/STAT signalling. Nat Biomed Eng. 2023. 10.1038/s41551-023-01143-w PMC11587785 38036617

[69] Sockolosky JT, Trotta E, Parisi G, Picton L, Su LL, Le AC, et al. Selective targeting of engineered T cells using orthogonal IL-2 cytokine-receptor complexes. Science. 2018;359(6379):1037–42. 29496879 10.1126/science.aar3246 PMC5947856

[70] Kloss CC, Lee J, Zhang A, Chen F, Melenhorst JJ, Lacey SF, et al. Dominant-negative TGF-β receptor enhances PSMA-Targeted human CAR T cell proliferation and augments prostate Cancer eradication. Mol Ther. 2018;26(7):1855–66. 29807781 10.1016/j.ymthe.2018.05.003 PMC6037129

[71] Smole A, Benton A, Poussin MA, Eiva MA, Mezzanotte C, Camisa B, et al. Expression of inducible factors reprograms CAR-T cells for enhanced function and safety. Cancer Cell. 2022;40(12):1470–e877. 36513049 10.1016/j.ccell.2022.11.006 PMC10367115

[72] Tang L, Pan S, Wei X, Xu X, Wei Q. Arming CAR-T cells with cytokines and more: innovations in the fourth-generation CAR-T development. Mol Ther. 2023;31(11):3146–62. 37803832 10.1016/j.ymthe.2023.09.021 PMC10638038

[73] Fang J, Ding N, Guo X, Sun Y, Zhang Z, Xie B et al. αPD-1-mesoCAR-T cells partially inhibit the growth of advanced/refractory ovarian cancer in a patient along with daily apatinib. J Immunother Cancer. 2021; 9(2). 10.1136/jitc-2020-001162 PMC7887368 33589520

[74] Rafiq S, Yeku OO, Jackson HJ, Purdon TJ, van Leeuwen DG, Drakes DJ, et al. Targeted delivery of a PD-1-blocking scFv by CAR-T cells enhances anti-tumor efficacy in vivo. Nat Biotechnol. 2018;36(9):847–56. 30102295 10.1038/nbt.4195 PMC6126939

[75] Zhang W, Liu L, Su H, Liu Q, Shen J, Dai H, et al. Chimeric antigen receptor macrophage therapy for breast tumours mediated by targeting the tumour extracellular matrix. Br J Cancer. 2019;121(10):837–45. 31570753 10.1038/s41416-019-0578-3 PMC6889154

[76] Nagarsheth N, Wicha MS, Zou W. Chemokines in the cancer microenvironment and their relevance in cancer immunotherapy. Nat Rev Immunol. 2017;17(9):559–72. 28555670 10.1038/nri.2017.49 PMC5731833

[77] Liu G, Rui W, Zhao X, Lin X. Enhancing CAR-T cell efficacy in solid tumors by targeting the tumor microenvironment. Cell Mol Immunol. 2021;18(5):1085–95. 33785843 10.1038/s41423-021-00655-2 PMC8093220

[78] Cadilha BL, Benmebarek MR, Dorman K, Oner A, Lorenzini T, Obeck H et al. Combined tumor-directed recruitment and protection from immune suppression enable CAR T cell efficacy in solid tumors. Sci Adv. 2021; 7(24). 10.1126/sciadv.abi5781 PMC8189699 34108220

[79] Pang N, Shi J, Qin L, Chen A, Tang Y, Yang H, et al. IL-7 and CCL19-secreting CAR-T cell therapy for tumors with positive glypican-3 or mesothelin. J Hematol Oncol. 2021;14(1):118. 34325726 10.1186/s13045-021-01128-9 PMC8323212

[80] Binnewies M, Roberts EW, Kersten K, Chan V, Fearon DF, Merad M, et al. Understanding the tumor immune microenvironment (TIME) for effective therapy. Nat Med. 2018;24(5):541–50. 29686425 10.1038/s41591-018-0014-x PMC5998822

[81] Johnson LR, Lee DY, Eacret JS, Ye D, June CH, Minn AJ. The immunostimulatory RNA RN7SL1 enables CAR-T cells to enhance autonomous and endogenous immune function. Cell. 2021;184(19):4981–e9514. 34464586 10.1016/j.cell.2021.08.004 PMC11338632

[82] Narayan V, Barber-Rotenberg JS, Jung IY, Lacey SF, Rech AJ, Davis MM, et al. PSMA-targeting TGFβ-insensitive armored CAR T cells in metastatic castration-resistant prostate cancer: a phase 1 trial. Nat Med. 2022;28(4):724–34. 35314843 10.1038/s41591-022-01726-1 PMC10308799

[83] Hu B, Ren J, Luo Y, Keith B, Young RM, Scholler J, et al. Augmentation of Antitumor immunity by human and mouse CAR T cells secreting IL-18. Cell Rep. 2017;20(13):3025–33. 28954221 10.1016/j.celrep.2017.09.002 PMC6002762

[84] Chmielewski M, Kopecky C, Hombach AA, Abken H. IL-12 release by engineered T cells expressing chimeric antigen receptors can effectively Muster an antigen-independent macrophage response on tumor cells that have shut down tumor antigen expression. Cancer Res. 2011;71(17):5697–706. 21742772 10.1158/0008-5472.CAN-11-0103

[85] Liu C, Chu D, Kalantar-Zadeh K, George J, Young HA, Liu G. Cytokines: from clinical significance to quantification. Adv Sci. 2021;8(15):e2004433. 10.1002/advs.202004433 PMC8336501 34114369

[86] Olivera I, Bolaños E, Gonzalez-Gomariz J, Hervas-Stubbs S, Mariño KV, Luri-Rey C, et al. mRNAs encoding IL-12 and a decoy-resistant variant of IL-18 synergize to engineer T cells for efficacious intratumoral adoptive immunotherapy. Cell Rep Med. 2023;4(3):100978. 36933554 10.1016/j.xcrm.2023.100978 PMC10040457

[87] Liu L, Bi E, Ma X, Xiong W, Qian J, Ye L, et al. Enhanced CAR-T activity against established tumors by polarizing human T cells to secrete interleukin-9. Nat Commun. 2020;11(1):5902. 33214555 10.1038/s41467-020-19672-2 PMC7677397

[88] Kim MY, Jayasinghe R, Devenport JM, Ritchey JK, Rettig MP, O’Neal J, et al. A long-acting interleukin-7, rhIL-7-hyFc, enhances CAR T cell expansion, persistence, and anti-tumor activity. Nat Commun. 2022;13(1):3296. 35697686 10.1038/s41467-022-30860-0 PMC9192727

[89] Liang S, Zheng R, Zuo B, Li J, Wang Y, Han Y, et al. SMAD7 expression in CAR-T cells improves persistence and safety for solid tumors. Cell Mol Immunol. 2024;21(3):213–26. 38177245 10.1038/s41423-023-01120-y PMC10901810

[90] Vincent RL, Gurbatri CR, Li F, Vardoshvili A, Coker C, Im J, et al. Probiotic-guided CAR-T cells for solid tumor targeting. Science. 2023;382(6667):211–18. 37824640 10.1126/science.add7034 PMC10915968

[91] Darowski D, Kobold S, Jost C, Klein C. Combining the best of two worlds: highly flexible chimeric antigen receptor adaptor molecules (CAR-adaptors) for the recruitment of chimeric antigen receptor T cells. MAbs. 2019;11(4):621–31. 30892136 10.1080/19420862.2019.1596511 PMC6601549

[92] Roybal KT, Rupp LJ, Morsut L, Walker WJ, McNally KA, Park JS, et al. Precision Tumor Recognition by T cells with Combinatorial Antigen-Sensing circuits. Cell. 2016;164(4):770–9. 26830879 10.1016/j.cell.2016.01.011 PMC4752902

[93] Allen GM, Frankel NW, Reddy NR, Bhargava HK, Yoshida MA, Stark SR, et al. Synthetic cytokine circuits that drive T cells into immune-excluded tumors. Science. 2022;378(6625):eaba1624. 36520915 10.1126/science.aba1624 PMC9970000

[94] Ruffo E, Butchy AA, Tivon Y, So V, Kvorjak M, Parikh A, et al. Post-translational covalent assembly of CAR and synNotch receptors for programmable antigen targeting. Nat Commun. 2023;14(1):2463. 37160880 10.1038/s41467-023-37863-5 PMC10169838

[95] Larson RC, Kann MC, Graham C, Mount CW, Castano AP, Lee WH, et al. Anti-TACI single and dual-targeting CAR T cells overcome BCMA antigen loss in multiple myeloma. Nat Commun. 2023;14(1):7509. 37980341 10.1038/s41467-023-43416-7 PMC10657357

[96] Schmidts A, Srivastava AA, Ramapriyan R, Bailey SR, Bouffard AA, Cahill DP, et al. Tandem chimeric antigen receptor (CAR) T cells targeting EGFRvIII and IL-13Rα2 are effective against heterogeneous glioblastoma. Neurooncol Adv. 2023;5(1):vdac185. 36751672 10.1093/noajnl/vdac185 PMC9896600

[97] Grada Z, Hegde M, Byrd T, Shaffer DR, Ghazi A, Brawley VS, et al. TanCAR: a novel bispecific chimeric Antigen receptor for Cancer Immunotherapy. Mol Ther Nucleic Acids. 2013;2(7):e105. 23839099 10.1038/mtna.2013.32 PMC3731887

[98] Bielamowicz K, Fousek K, Byrd TT, Samaha H, Mukherjee M, Aware N, et al. Trivalent CAR T cells overcome interpatient antigenic variability in glioblastoma. Neuro Oncol. 2018;20(4):506–18. 29016929 10.1093/neuonc/nox182 PMC5909636

[99] Hirabayashi K, Du H, Xu Y, Shou P, Zhou X, Fucá G, et al. Dual targeting CAR-T cells with optimal Costimulation and Metabolic Fitness enhance Antitumor Activity and prevent escape in solid tumors. Nat Cancer. 2021;2(9):904–18. 34746799 10.1038/s43018-021-00244-2 PMC8570569

[100] Labanieh L, Majzner RG, Mackall CL. Programming CAR-T cells to kill cancer. Nat Biomed Eng. 2018;2(6):377–91. 31011197 10.1038/s41551-018-0235-9

[101] DeNardo DG, Ruffell B. Macrophages as regulators of tumour immunity and immunotherapy. Nat Rev Immunol. 2019;19(6):369–82. 30718830 10.1038/s41577-019-0127-6 PMC7339861

[102] Christofides A, Strauss L, Yeo A, Cao C, Charest A, Boussiotis VA. The complex role of tumor-infiltrating macrophages. Nat Immunol. 2022;23(8):1148–56. 35879449 10.1038/s41590-022-01267-2 PMC10754321

[103] Bied M, Ho WW, Ginhoux F, Blériot C. Roles of macrophages in tumor development: a spatiotemporal perspective. Cell Mol Immunol. 2023;20(9):983–92. 37429944 10.1038/s41423-023-01061-6 PMC10468537

[104] Li X, Zhang Y, Li S, Shi J, Liu C, Li X et al. Macrophage hitchhiking for systematic suppression in postablative multifocal HCC. Hepatology. 2024. 10.1097/HEP.0000000000000903 38683582

[105] Xiang X, Wang J, Lu D, Xu X. Targeting tumor-associated macrophages to synergize tumor immunotherapy. Signal Transduct Target Ther. 2021;6(1):75. 33619259 10.1038/s41392-021-00484-9 PMC7900181

[106] Perdiguero EG, Geissmann F. The development and maintenance of resident macrophages. Nat Immunol. 2016;17(1):2–8. 26681456 10.1038/ni.3341 PMC4950995

[107] Xiang C, Li H, Tang W. Targeting CSF-1R represents an effective strategy in modulating inflammatory diseases. Pharmacol Res. 2023; 187(106566. 10.1016/j.phrs.2022.106566 36423789

[108] Zhang P, Zhao S, Wu C, Li J, Li Z, Wen C, et al. Effects of CSF1R-targeted chimeric antigen receptor-modified NK92MI & T cells on tumor-associated macrophages. Immunotherapy. 2018;10(11):935–49. 30149762 10.2217/imt-2018-0012

[109] Shao Z, Tan Y, Shen Q, Hou L, Yao B, Qin J, et al. Molecular insights into ligand recognition and activation of chemokine receptors CCR2 and CCR3. Cell Discov. 2022;8(1):44. 35570218 10.1038/s41421-022-00403-4 PMC9108096

[110] Hao Q, Vadgama JV, Wang P. CCL2/CCR2 signaling in cancer pathogenesis. Cell Commun Signal. 2020;18(1):82. 32471499 10.1186/s12964-020-00589-8 PMC7257158

[111] Qian BZ, Li J, Zhang H, Kitamura T, Zhang J, Campion LR, et al. CCL2 recruits inflammatory monocytes to facilitate breast-tumour metastasis. Nature. 2011;475(7355):222–5. 21654748 10.1038/nature10138 PMC3208506

[112] Li H, Harrison EB, Li H, Hirabayashi K, Chen J, Li QX, et al. Targeting brain lesions of non-small cell lung cancer by enhancing CCL2-mediated CAR-T cell migration. Nat Commun. 2022;13(1):2154. 35443752 10.1038/s41467-022-29647-0 PMC9021299

[113] Feng M, Jiang W, Kim BYS, Zhang CC, Fu YX, Weissman IL. Phagocytosis checkpoints as new targets for cancer immunotherapy. Nat Rev Cancer. 2019;19(10):568–86. 31462760 10.1038/s41568-019-0183-z PMC7002027

[114] Matlung HL, Szilagyi K, Barclay NA, van den Berg TK. The CD47-SIRPα signaling axis as an innate immune checkpoint in cancer. Immunol Rev. 2017;276(1):145–64. 28258703 10.1111/imr.12527

[115] Zhang W, Zeng Y, Xiao Q, Wu Y, Liu J, Wang H, et al. An in-situ peptide-antibody self-assembly to block CD47 and CD24 signaling enhances macrophage-mediated phagocytosis and anti-tumor immune responses. Nat Commun. 2024;15(1):5670. 38971872 10.1038/s41467-024-49825-6 PMC11227529

[116] Chen H, Yang Y, Deng Y, Wei F, Zhao Q, Liu Y et al. Delivery of CD47 blocker SIRPα-Fc by CAR-T cells enhances antitumor efficacy. J Immunother Cancer. 2022; 10(2). 10.1136/jitc-2021-003737 PMC8811602 35110357

[117] Deczkowska A, Weiner A, Amit I. The Physiology, Pathology, and potential therapeutic applications of the TREM2 signaling pathway. Cell. 2020;181(6):1207–17. 32531244 10.1016/j.cell.2020.05.003

[118] Morad G, Helmink BA, Sharma P, Wargo JA. Hallmarks of response, resistance, and toxicity to immune checkpoint blockade. Cell. 2021;184(21):5309–37. 34624224 10.1016/j.cell.2021.09.020 PMC8767569

[119] Fife BT, Bluestone JA. Control of peripheral T-cell tolerance and autoimmunity via the CTLA-4 and PD-1 pathways. Immunol Rev. 2008; 224(166 – 82. 10.1111/j.1600-065X.2008.00662.x 18759926

[120] He X, Xu C. Immune checkpoint signaling and cancer immunotherapy. Cell Res. 2020;30(8):660–69. 32467592 10.1038/s41422-020-0343-4 PMC7395714

[121] Liu Y, Zheng P. Preserving the CTLA-4 checkpoint for Safer and more effective Cancer immunotherapy. Trends Pharmacol Sci. 2020;41(1):4–12. 31836191 10.1016/j.tips.2019.11.003 PMC7210725

[122] Tai X, Van Laethem F, Pobezinsky L, Guinter T, Sharrow SO, Adams A, et al. Basis of CTLA-4 function in regulatory and conventional CD4(+) T cells. Blood. 2012;119(22):5155–63. 22403258 10.1182/blood-2011-11-388918 PMC3369608

[123] Lin S, Cheng L, Ye W, Li S, Zheng D, Qin L et al. Chimeric CTLA4-CD28-CD3z T Cells Potentiate Antitumor Activity Against CD80/CD86-Positive B Cell Malignancies. Front Immunol. 2021; 12(642528. 10.3389/fimmu.2021.642528 PMC8050336 33868277

[124] Zhou X, Cao H, Fang SY, Chow RD, Tang K, Majety M, et al. CTLA-4 tail fusion enhances CAR-T antitumor immunity. Nat Immunol. 2023;24(9):1499–510. 37500885 10.1038/s41590-023-01571-5 PMC11344484

[125] He X, Xu C. PD-1: a driver or passenger of T cell exhaustion? Mol Cell. 2020;77(5):930–31. 32142689 10.1016/j.molcel.2020.02.013

[126] Anderson AC, Joller N, Kuchroo VK, Lag-3. Tim-3, and TIGIT: co-inhibitory receptors with Specialized functions in Immune Regulation. Immunity. 2016;44(5):989–1004. 27192565 10.1016/j.immuni.2016.05.001 PMC4942846

[127] Kandel S, Adhikary P, Li G, Cheng K. The TIM3/Gal9 signaling pathway: an emerging target for cancer immunotherapy. Cancer Lett. 2021;510:67–78. 33895262 10.1016/j.canlet.2021.04.011 PMC8168453

[128] Jafarzadeh L, Masoumi E, Mirzaei HR, Alishah K, Fallah-Mehrjardi K, Khakpoor-Koosheh M, et al. Targeted knockdown of Tim3 by short hairpin RNAs improves the function of anti-mesothelin CAR T cells. Mol Immunol. 2021;139:1–9. 34450537 10.1016/j.molimm.2021.06.007

[129] Chen L, Flies DB. Molecular mechanisms of T cell co-stimulation and co-inhibition. Nat Rev Immunol. 2013;13(4):227–42. 23470321 10.1038/nri3405 PMC3786574

[130] Yang F, Zhang F, Ji F, Chen J, Li J, Chen Z et al. Self-delivery of TIGIT-blocking scFv enhances CAR-T immunotherapy in solid tumors. Front Immunol. 2023; 14(1175920. 10.3389/fimmu.2023.1175920 PMC10287952 37359558

[131] Sun WZ, Lin HW, Chen WY, Chien CL, Lai YL, Chen J et al. Dual inhibition of BTLA and PD-1 can enhance therapeutic efficacy of paclitaxel on intraperitoneally disseminated tumors. J Immunother Cancer. 2023; 11(7). 10.1136/jitc-2023-006694 PMC10357656 37463789

[132] Liu W, Chou TF, Garrett-Thomson SC, Seo GY, Fedorov E, Ramagopal UA et al. HVEM structures and mutants reveal distinct functions of binding to LIGHT and BTLA/CD160. J Exp Med. 2021; 218(12). 10.1084/jem.20211112 PMC8558838 34709351

[133] Guruprasad P, Carturan A, Zhang Y, Cho JH, Kumashie KG, Patel RP, et al. The BTLA-HVEM axis restricts CAR T cell efficacy in cancer. Nat Immunol. 2024;25(6):1020–32. 38831106 10.1038/s41590-024-01847-4

[134] Qi Y, Liu B, Sun Q, Xiong X, Chen Q. Immune Checkpoint targeted therapy in glioma: status and hopes. Front Immunol. 2020;11:578877. 33329549 10.3389/fimmu.2020.578877 PMC7729019

[135] Brandt CS, Baratin M, Yi EC, Kennedy J, Gao Z, Fox B, et al. The B7 family member B7-H6 is a tumor cell ligand for the activating natural killer cell receptor NKp30 in humans. J Exp Med. 2009;206(7):1495–503. 19528259 10.1084/jem.20090681 PMC2715080

[136] Gacerez AT, Sentman CL. T-bet promotes potent antitumor activity of CD4(+) CAR T cells. Cancer Gene Ther. 2018;25(5–6):117–28. 29515240 10.1038/s41417-018-0012-7 PMC6021366

[137] Delgoffe GM, Woo SR, Turnis ME, Gravano DM, Guy C, Overacre AE, et al. Stability and function of regulatory T cells is maintained by a neuropilin-1-semaphorin-4a axis. Nature. 2013;501(7466):252–6. 23913274 10.1038/nature12428 PMC3867145

[138] Liu C, Somasundaram A, Manne S, Gocher AM, Szymczak-Workman AL, Vignali KM, et al. Neuropilin-1 is a T cell memory checkpoint limiting long-term antitumor immunity. Nat Immunol. 2020;21(9):1010–21. 32661362 10.1038/s41590-020-0733-2 PMC7442600

[139] Klepsch V, Gerner RR, Klepsch S, Olson WJ, Tilg H, Moschen AR, et al. Nuclear orphan receptor NR2F6 as a safeguard against experimental murine colitis. Gut. 2018;67(8):1434–44. 28779026 10.1136/gutjnl-2016-313466 PMC6204953

[140] Klepsch V, Pommermayr M, Humer D, Brigo N, Hermann-Kleiter N, Baier G. Targeting the orphan nuclear receptor NR2F6 in T cells primes tumors for immune checkpoint therapy. Cell Commun Signal. 2020;18(1):8. 31937317 10.1186/s12964-019-0454-z PMC6961368

[141] Sabado RL, Balan S, Bhardwaj N. Dendritic cell-based immunotherapy. Cell Res. 2017;27(1):74–95. 28025976 10.1038/cr.2016.157 PMC5223236

[142] See P, Dutertre CA, Chen J, Günther P, McGovern N, Irac SE et al. Mapping the human DC lineage through the integration of high-dimensional techniques. Science. 2017; 356(6342). 10.1126/science.aag3009 PMC7611082 28473638

[143] Ferris ST, Durai V, Wu R, Theisen DJ, Ward JP, Bern MD, et al. cDC1 prime and are licensed by CD4(+) T cells to induce anti-tumour immunity. Nature. 2020;584(7822):624–29. 32788723 10.1038/s41586-020-2611-3 PMC7469755

[144] Anandasabapathy N, Victora GD, Meredith M, Feder R, Dong B, Kluger C, et al. Flt3L controls the development of radiosensitive dendritic cells in the meninges and choroid plexus of the steady-state mouse brain. J Exp Med. 2011;208(8):1695–705. 21788405 10.1084/jem.20102657 PMC3149213

[145] Lai J, Mardiana S, House IG, Sek K, Henderson MA, Giuffrida L, et al. Adoptive cellular therapy with T cells expressing the dendritic cell growth factor Flt3L drives epitope spreading and antitumor immunity. Nat Immunol. 2020;21(8):914–26. 32424363 10.1038/s41590-020-0676-7

[146] Richards DM, Sefrin JP, Gieffers C, Hill O, Merz C. Concepts for agonistic targeting of CD40 in immuno-oncology. Hum Vaccin Immunother. 2020;16(2):377–87. 31403344 10.1080/21645515.2019.1653744 PMC7062441

[147] Vonderheide RH. The Immune Revolution: a case for Priming, not checkpoint. Cancer Cell. 2018;33(4):563–69. 29634944 10.1016/j.ccell.2018.03.008 PMC5898647

[148] Kuhn NF, Purdon TJ, van Leeuwen DG, Lopez AV, Curran KJ, Daniyan AF, et al. CD40 Ligand-Modified Chimeric Antigen Receptor T Cells Enhance Antitumor Function by eliciting an endogenous Antitumor response. Cancer Cell. 2019;35(3):473–e886. 30889381 10.1016/j.ccell.2019.02.006 PMC6428219

[149] Zhang Y, Wang P, Wang T, Fang Y, Ding Y, Qian Q. Chimeric antigen receptor T cells engineered to secrete CD40 agonist antibodies enhance antitumor efficacy. J Transl Med. 2021;19(1):82. 33602263 10.1186/s12967-021-02750-4 PMC7890961

[150] De Palma M, Hanahan D. Milestones in tumor vascularization and its therapeutic targeting. Nat Cancer. 2024;5(6):827–43. 38918437 10.1038/s43018-024-00780-7

[151] Schito L, Rey S, Hypoxia. Turning vessels into vassals of cancer immunotolerance. Cancer Lett. 2020;487:74–84. 32470491 10.1016/j.canlet.2020.05.015

[152] Schaaf MB, Garg AD, Agostinis P. Defining the role of the tumor vasculature in antitumor immunity and immunotherapy. Cell Death Dis. 2018;9(2):115. 29371595 10.1038/s41419-017-0061-0 PMC5833710

[153] Akbari P, Katsarou A, Daghighian R, van Mil L, Huijbers EJM, Griffioen AW, et al. Directing CAR T cells towards the tumor vasculature for the treatment of solid tumors. Biochim Biophys Acta Rev Cancer. 2022;1877(3):188701. 35202772 10.1016/j.bbcan.2022.188701

[154] Yang Y, Cao Y. The impact of VEGF on cancer metastasis and systemic disease. Semin Cancer Biol. 2022;86(Pt 3):251–61. 35307547 10.1016/j.semcancer.2022.03.011

[155] Apte RS, Chen DS, Ferrara N. VEGF in Signaling and Disease: Beyond Discovery and Development. Cell. 2019;176(6):1248–64. 30849371 10.1016/j.cell.2019.01.021 PMC6410740

[156] Ferrara N, Gerber HP, LeCouter J. The biology of VEGF and its receptors. Nat Med. 2003;9(6):669–76. 12778165 10.1038/nm0603-669

[157] Wang W, Ma Y, Li J, Shi HS, Wang LQ, Guo FC, et al. Specificity redirection by CAR with human VEGFR-1 affinity endows T lymphocytes with tumor-killing ability and anti-angiogenic potency. Gene Ther. 2013;20(10):970–8. 23636245 10.1038/gt.2013.19

[158] Xing H, Yang X, Xu Y, Tang K, Tian Z, Chen Z, et al. Anti-tumor effects of vascular endothelial growth factor/vascular endothelial growth factor receptor binding domain-modified chimeric antigen receptor T cells. Cytotherapy. 2021;23(9):810–19. 34244079 10.1016/j.jcyt.2021.05.008

[159] Xie Y, Su N, Yang J, Tan Q, Huang S, Jin M, et al. FGF/FGFR signaling in health and disease. Signal Transduct Target Ther. 2020;5(1):181. 32879300 10.1038/s41392-020-00222-7 PMC7468161

[160] Katoh M, Loriot Y, Brandi G, Tavolari S, Wainberg ZA, Katoh M. FGFR-targeted therapeutics: clinical activity, mechanisms of resistance and new directions. Nat Rev Clin Oncol. 2024. 10.1038/s41571-024-00869-z 38424198

[161] Zhang P, Yue L, Leng Q, Chang C, Gan C, Ye T, et al. Targeting FGFR for cancer therapy. J Hematol Oncol. 2024;17(1):39. 38831455 10.1186/s13045-024-01558-1 PMC11149307

[162] Tian M, Wei JS, Shivaprasad N, Highfill SL, Gryder BE, Milewski D, et al. Preclinical development of a chimeric antigen receptor T cell therapy targeting FGFR4 in rhabdomyosarcoma. Cell Rep Med. 2023;4(10):101212. 37774704 10.1016/j.xcrm.2023.101212 PMC10591056

[163] Sutherland TE, Dyer DP, Allen JE. The extracellular matrix and the immune system: a mutually dependent relationship. Science. 2023;379(6633):eabp8964. 36795835 10.1126/science.abp8964

[164] Martin JD, Cabral H, Stylianopoulos T, Jain RK. Improving cancer immunotherapy using nanomedicines: progress, opportunities and challenges. Nat Rev Clin Oncol. 2020;17(4):251–66. 32034288 10.1038/s41571-019-0308-z PMC8272676

[165] Liu Y, Zhang X, Gu W, Su H, Wang X, Wang X et al. Unlocking the crucial role of cancer-associated fibroblasts in tumor metastasis: mechanisms and therapeutic prospects. J Adv Res. 2024. 10.1016/j.jare.2024.05.031 PMC12126706 38825314

[166] Kieffer Y, Hocine HR, Gentric G, Pelon F, Bernard C, Bourachot B, et al. Single-cell analysis reveals fibroblast clusters linked to Immunotherapy Resistance in Cancer. Cancer Discov. 2020;10(9):1330–51. 32434947 10.1158/2159-8290.CD-19-1384

[167] Xiao Z, Todd L, Huang L, Noguera-Ortega E, Lu Z, Huang L, et al. Desmoplastic stroma restricts T cell extravasation and mediates immune exclusion and immunosuppression in solid tumors. Nat Commun. 2023;14(1):5110. 37607999 10.1038/s41467-023-40850-5 PMC10444764

[168] Massagué J, Sheppard D. TGF-β signaling in health and disease. Cell. 2023;186(19):4007–37. 37714133 10.1016/j.cell.2023.07.036 PMC10772989

[169] Derynck R, Turley SJ, Akhurst RJ. TGFβ biology in cancer progression and immunotherapy. Nat Rev Clin Oncol. 2021;18(1):9–34. 32710082 10.1038/s41571-020-0403-1 PMC9721352

[170] Chen IX, Chauhan VP, Posada J, Ng MR, Wu MW, Adstamongkonkul P, et al. Blocking CXCR4 alleviates desmoplasia, increases T-lymphocyte infiltration, and improves immunotherapy in metastatic breast cancer. Proc Natl Acad Sci U S A. 2019;116(10):4558–66. 30700545 10.1073/pnas.1815515116 PMC6410779

[171] Sun R, Sun Y, Wu C, Liu Y, Zhou M, Dong Y, et al. CXCR4-modified CAR-T cells suppresses MDSCs recruitment via STAT3/NF-κB/SDF-1α axis to enhance efficacy against pancreatic cancer. Mol Ther. 2023;31(11):3193–209. 37735875 10.1016/j.ymthe.2023.09.010 PMC10638076

[172] Allen GM, Lim WA. Rethinking cancer targeting strategies in the era of smart cell therapeutics. Nat Rev Cancer. 2022;22(12):693–702. 36175644 10.1038/s41568-022-00505-x

